# Proteomic Analysis of Urine to Identify Breast Cancer Biomarker Candidates Using a Label-Free LC-MS/MS Approach

**DOI:** 10.1371/journal.pone.0141876

**Published:** 2015-11-06

**Authors:** Julia Beretov, Valerie C. Wasinger, Ewan K. A. Millar, Peter Schwartz, Peter H. Graham, Yong Li

**Affiliations:** 1 Cancer Care Centre, St George Hospital, Kogarah, Australia; 2 St George and Sutherland Clinical School, Faculty of Medicine, University of New South Wales (UNSW), Kensington, Australia; 3 SEALS, Anatomical Pathology, St George Hospital, Kogarah, Australia; 4 Bioanalytical Mass Spectrometry Facility, Mark Wainwright Analytical Centre, UNSW, Kensington, Australia; 5 School of Medical Sciences, UNSW, Kensington, Australia; 6 Cancer Research Program, Kinghorn Cancer Centre and Garvan Institute of Medical Research, Darlinghurst, Australia; 7 School of Medicine and Health Sciences, University of Western Sydney, Campbelltown, Australia; 8 Breast Surgery, St George Private Hospital, Kogarah, Australia; King Faisal Specialist Hospital & Research center, SAUDI ARABIA

## Abstract

**Introduction:**

Breast cancer is a complex heterogeneous disease and is a leading cause of death in women. Early diagnosis and monitoring progression of breast cancer are important for improving prognosis. The aim of this study was to identify protein biomarkers in urine for early screening detection and monitoring invasive breast cancer progression.

**Method:**

We performed a comparative proteomic analysis using ion count relative quantification label free LC-MS/MS analysis of urine from breast cancer patients (n = 20) and healthy control women (n = 20).

**Results:**

Unbiased label free LC-MS/MS-based proteomics was used to provide a profile of abundant proteins in the biological system of breast cancer patients. Data analysis revealed 59 urinary proteins that were significantly different in breast cancer patients compared to the normal control subjects (*p*<0.05, fold change >3). Thirty-six urinary proteins were exclusively found in specific breast cancer stages, with 24 increasing and 12 decreasing in their abundance. Amongst the 59 significant urinary proteins identified, a list of 13 novel up-regulated proteins were revealed that may be used to detect breast cancer. These include stage specific markers associated with pre-invasive breast cancer in the ductal carcinoma in-situ (DCIS) samples (Leucine LRC36, MAST4 and Uncharacterized protein CI131), early invasive breast cancer (DYH8, HBA, PEPA, uncharacterized protein C4orf14 (CD014), filaggrin and MMRN2) and metastatic breast cancer (AGRIN, NEGR1, FIBA and Keratin KIC10). Preliminary validation of 3 potential markers (ECM1, MAST4 and filaggrin) identified was performed in breast cancer cell lines by Western blotting. One potential marker MAST4 was further validated in human breast cancer tissues as well as individual human breast cancer urine samples with immunohistochemistry and Western blotting, respectively.

**Conclusions:**

Our results indicate that urine is a useful non-invasive source of biomarkers and the profile patterns (biomarkers) identified, have potential for clinical use in the detection of BC. Validation with a larger independent cohort of patients is required in the following study.

## Introduction

Breast cancer (BC) is a major public health problem worldwide. Despite the widespread use of mammographic screening, which has contributed to reduced mortality, BC is still the most common form of cancer among women. It can only be detected using mammography if there is a visible, detectible abnormality with architectural distortion or calcification, which correlates with the presence of several hundred thousand tumor cells. Once BC has been biopsied and the diagnosis has been confirmed pathologically, the tumor is surgically excised. The complexity and heterogeneity of individual tumors play an important role in therapeutic decision making. Pathological examination is still the gold standard for diagnosis and assessment of prognostic indicators in BC which include tumor size, grade (degree of tumor cell differentiation), presence or absence of positive lymph nodes (metastases), immunohistochemical expression of key proteins such as estrogen receptor (ER), progesterone receptor (PR) and HER2 [1].

Although advances in BC diagnosis have been made in the last decade, there are still many BC patients who cannot be diagnosed in the early stages of disease or monitored adequately for tumor recurrence using current techniques. To reduce morbidity and mortality from BC, novel approaches must be considered for screening, early detection and prevention, as well as for monitoring cancer progression or recurrence. The early detection of ductal carcinoma in-situ (DCIS) or invasive breast cancer (IBC) may prevent the development of life threatening metastatic disease. Additionally, monitoring metastatic progression could identify early BC recurrence and help guide therapeutic decision making.

Human urine is one of the most interesting and useful bio-fluids for clinical proteomics studies. Advances in proteomics, especially in mass spectrometry (MS) [2,3] have rapidly changed our knowledge of urine proteins which have simultaneously led to the identification and quantification of thousands of unique proteins and peptides in a complex biological fluid [4,5]. Proteomic studies of urine are highly informative, and have been successfully used to discover novel markers for cancer diagnosis and surveillance [6–9] as well as for monitoring cancer progression [10,11]. Technological development combined with the addition of urine screening would increase the knowledge about patient status and further assist assessment and treatment in clinical practice. Proteomic analysis of urine holds the potential to apply a non-invasive method to identify novel biomarkers of BC. However, investigation of urinary proteins from different stages of BC patients using a liquid chromatography tandem mass spectrometry (LC-MS/MS) proteomic approach has not been reported to date.

In this study, we used a label free LC-MS/MS technique to test the feasibility of urine as a source for BC biomarkers and identify the urinary proteins for BC diagnosis and monitoring progression. One potential marker (extracellular matrix protein 1 (ECM1) previously identified and associated with BC), and two novel potential protein markers (MAST4-microtubule associated serine/threonine kinase family member 4 and filaggrin) identified from BC urine were validated in BC cell lines and MAST4 was validated in a small number of primary BC tissues and in the individual human BC urine samples, demonstrating the link of these proteins with BC. However, a larger cohort of BC patients’ samples is needed for the validation of the identified potential markers in the following studies. The proteins identified showed significant differences in abundance between the different BC disease stages which provide a useful reservoir of biomarkers for the detection of early and advanced BC.

## Materials and Methods

### Study design and ethics

In this pilot study, all the female BC subjects had received detailed diagnostic procedures, i.e. a physical breast examination, mammography, ultrasound and biopsy or excision with a detailed pathological report on the cancer. Ethics approval for the collection of human urine and tissue samples was approved by the South Eastern Sydney Area Health Service Ethics Committee (SEA HRCE) (#07/71Li). The study was designed and conducted in accordance with the ethical principles and all participants signed informed consent forms. None of the subjects had received any prior treatment, either endocrine or chemotherapy. The healthy disease free control group (n = 20) were age matched with the BC patients (range 35–70 years, mean age, 51 ± 10.5 years). Urine samples were collected prior to surgery while BC tissues and normal part of breast tissues were collected after surgery (St George Private Hospital, Sydney, Australia). The collected samples were evaluated and grouped in the analysis according to histopathology report, after diagnosis. The breast carcinoma typing and grading were performed by a pathologist according to the World Health Organization criteria [1]. The samples were grouped into 3 different BC stages: DCIS (n = 6), early IBC (with or without axillary lymph node involvement, but no distant metastases, n = 8), and metastatic breast cancer (MBC) (distant metastases to viscera or bone, n = 6) along with a group of samples with benign breast disease (BBD) (n = 6). The histopathology characteristics and clinical features are summarized in Table 1.

**Table 1:** The histopathology characteristics and parameters, of the patients in this study.

*Patient Groups*	*Number of Patients*	*Tumour Size (mm)*	*TumourGrade*	*Histological Diagnosis*	*DCIS*	*LN +/-*	*Biomarkers ER/PR status*	*HER-2*
**DCIS**	6	25–48	3	DCIS				
**IBC**	5	9–32	2 or 3	Ductal or Lobular	present	+	+/+	-
			2 or 3	Ductal or Lobular	present	-	+/-	-
	3	14–22						
**MBC**	6	10–23	2 or 3	Ductal or Lobular	present	+	+/+	-
			2 or 3	Ductal or Lobular	present	+	+/-	+
		10–24						
**BBD**	6	-	-	Fibrocystic change, fibroadenoma	-	-	-	-

**Notes:**LN: Lymph Node involvement; +/-: positive involved/ negative not involved; Tumour grade 1–3 [1].

Abbreviations: DCIS: ductal carcinoma in-situ; IBC: invasive BC; MBC: metastatic BC; BBD; benign breast disease; ER: oestrogen receptor status; PR: progesterone receptor status; HER2: human epidermal growth factor receptor status.

### Urine sample collection and processing

Clean catch (no skin contamination), midstream 30–50 mL urine samples were collected in a sterile tube and immediately transported on ice. The urine was centrifuged at 2000 x *g* (4000 rpm), at 4°C for 10 min to remove insoluble materials and cellular debris. The supernatants were aliquoted and frozen at -20°C and then transferred to -80°C for long term storage. All samples were handled by the same standard operating procedures and processed for storage within one hour of collection.

All urine samples had protein concentration and urine creatinine levels measured, and abnormal samples were excluded from the study. The appropriate volume of urine samples was then pooled within the appropriate group to ensure the same total concentration of proteins for proteomics analysis. The pooled urine supernatants from each group were subjected to total protein precipitation by 1:8 sample-solvent ratio of ice-cold (-20°C) acetone, mixed and stored for 1 hour at −20°C, and then high speed centrifuged with high speed centrifugation (HSC), 11,000 x g at 4°C for 30 min. The supernatants were removed and the pellets were further air-dried.

To further precipitate and concentrate the proteins, the pellets were resuspended in 2 mL of fresh TCA solution (concentrated: 10 g TCA in 10 mL Milli-Q H_2_O) in a 4:1 sample-to-solvent ratio, vortexed, incubated at 4°C for 1 hour and then centrifuged with HSC at 4°C for 30 min. After carefully discarding the supernatants, protein pellets were washed twice with ice-cold acetone for 15 min, along with HSC at 4°C for 15 min. All pellets were air-dried as our published method [12].

All protein pellets were resuspended in 100 μL of rehydration buffer (RB) solution (2 M thiourea, 7 M urea, 40 mM Tris-base, 1% 3-[(3 cholamidopropyl) dimethylammonio]-1-propanesulfonate (CHAPS), 50 mM DTT and 0.1% Bromothymol Blue) before use, and vigorously vortexed to ensure the pellets were completely dissolved. The protein concentrations of samples were determined with 2-D Quant Kit method (GE healthcare-Life sciences. Product code 80-6483-56) following the manufacturer’s instructions.

### Urine sample protein clean-up and digestion

The peptide fractions were enzymatically digested with trypsin. Lyophilized protein samples were reconstituted with 25 μL of 50 mM Ammonium bi-carbonate (AMBIC) (pH 8). Trypsin (12.5 ng/μL trypsin proteomic grade, Sigma-Aldrich, St. Louis, MO, USA) was added to a final enzyme-to-protein ratio of 1:100 (w/w) and was incubated at 37°C overnight. The reaction was stopped by acidifying the preparation to ~pH 3 using neat formic acid (FA). Samples were dried in a vacuum centrifuge to concentrate the samples which were stored at -20°C. Following trypsin digestion, the peptide samples were purified using Strong Cation exchange (SCX) and C18 StageTips (Thermo Scientific, USA) following the manufacturer’s instructions.

### LC-MS/MS analysis of urine sample

Label-free LC-MS/MS quantification was performed using an Orbitrap Velos (LTQ-Orbitrap, Thermo Scientific, USA). All urine samples were run in triplicate. Peptides were reconstituted in 10 μL of 0.1% FA and separated by nano-LC using an Ultimate 3000 HPLC and auto sampler (Dionex, Amsterdam, Netherlands). The samples (0.6 μL, 2 μg total load) were loaded onto a micro C18 pre-column (500 μm × 2 mm, Michrom Bio-resources, Auburn, CA, USA) with Buffer A at 10 μL/min (2% ACN and 0.01% Heptafluorobutyric Acid (HFBA) in water). After a 4-min wash, the pre-column was switched (Valco 10 port valve, Dionex) into line with a fritless nano column (75 μm diameter × 12 cm) containing reverse phase C18 media (3 μm, 200Å Magic, Michrom Bio-resources). Peptides were eluted using a linear gradient of Buffer A to Buffer B (98% ACN, 0.01% HFBA in water) at 250 nL/min over 60 min. High voltage (2000 V) was applied to a low volume tee (Upchurch Scientific, Oak Harbor, WA, USA) and the column tip positioned ~0.5 cm from the heated capillary (T = 280°C) of an Orbitrap Velos (Thermo Electron, Bremen, Germany) mass spectrometer. Positive ions were generated by electrospray and the Orbitrap was operated in data-dependent acquisition mode. A survey scan MS was acquired in the Orbitrap in the 350–1750 *m/z* range with the resolution set to a value of 30 000 at *m/z =* 400 (with an accumulation target value of 1 000 000 ions), with lock-mass enabled. The 10 most intense ions (>5000 counts) with charge states +2 to +4 were sequentially isolated and fragmented within the linear ion trap using CID with an activation *q* = 0.25 and activation time of 30 milliseconds (ms) at a target value of 30 000 ions. The *m/z* ratios selected for MS/MS were dynamically excluded for 30 seconds to prevent repetitive selection of the same peptide.

### Label-free LC-MS quantitative profiling

MS peak intensities were analyzed using Progensis QI, LC-MS data analysis software (version 4.1, Nonlinear Dynamics, Newcastle upon Tyne, UK). Ion intensity maps from each run were aligned to a reference map and ion feature matching was achieved by aligning consistent ion *m/z* and retention times. The peptide intensities were normalized against total intensity (sample specific log-scale abundance ratio scaling factor) and compared between groups by one-way analysis of variance (ANOVA, *p* ≤ 0.05 for statistical significance). Type I errors were controlled by False Discovery Rate (FDR) with *q* value set at 0.02 [13,14].

MS/MS spectra were searched and identified against the human protein database Uni-Prot database (downloaded January 2013) using the database search program MASCOT (Matrix Science, London, UK, www.matrixscience.com). Parent and fragment ions were searched with tolerances of ± 6 ppm and ± 0.6 Da, respectively. Searched peptide charge states were limited to +2 to +4. Deamination (M), Oxidation. Phosphorylation was chosen as variable modifications. Only peptides with an ion score >25 were considered for protein identification. Proteins were considered to be significantly different at *p* < 0.05; fold change >3.

### Generation of the heat map

The area under curve (AUC) of all MS1 peaks generated from comparisons among different stages of BC in urine (Progensis data) was normalised to the mean of all AUC using TIBCo spotfire (Boston, MA, USA). The clustering method used is UPGMA and distance measure was Cosine correlation in logarithmic scale for rows. Columns were clustered using a Ward’s method with distance measured using Half Square Euclidean.

### Cell lines and cell culture

The human primary breast cancer cell line (BT-474) and metastatic cell lines (MDA-MB-231, MCF-7 and SK-BR-3) were obtained from the American Type Tissue Culture Collection (ATCC, Rockville, MD). All tissue culture reagents were supplied by Invitrogen Australia Pty Ltd (Melbourne, VIC, Australia), unless otherwise stated. The primary BC cell line (BT-474) was maintained in Improved Minimal Essential Medium (IMEM) supplemented with 10% heated-inactivated fetal bovine serum (FBS), 50 U/mL of penicillin and 50 μg/mL of streptomycin. Other three metastatic BC cell lines (MDA-MB-231, MCF-7 and SKBR-3) cell lines were cultured in RPMI-1640 supplemented with 10% FBS, 50 U/mL of penicillin and 50 μg/mL of streptomycin. All cell lines were maintained in a humidified incubator at 37°C and 5% CO_2_. Protein from each cell line was extracted. Briefly, cells with 80–90% confluency were rinsed twice with Dulbecco’s phosphate-buffered saline (DPBS) (pH7.2), and then lysed in a buffer containing 50 mmol/L Tris-HCl (pH 8.0), 150 mmol/L Sodium chloride (NaCl), 0.1% SDS, 10 mmol/L NaF, 1 mmol/L Sodium orthovanadate (Na3VO4), 0.5% sodium deoxycholate, 1% Triton X-100, and 1/12 (v/v) protease inhibitor cocktail (Sigma-Aldrich, Pty Ltd, Castle Hills, NSW, Australia). After a brief incubation for 5 min on ice, the lysates were collected and centrifuged at 14,000 rpm for 10 min at 4°C and the supernatants were collected and stored at -80°C for Western blotting experiment.

### Western blotting

Protein expression levels were determined by Western blotting analysis as previously described [15]. Human urine protein pellets (following acetone/TCA extraction) were re-suspended in rehydration buffer (25 mmol/L Tris-HCl (pH 8.0), 0.5% SDS). Briefly, total proteins were measured using a BCA assay kit (Thermo Scientific, Rockford, IL, USA). Proteins from human BC cell lines and human urine samples were resolved on 4–20% Tris-Nupage gels (Invitrogen Australia Pty Ltd, Melbourne, VIC, Australia) and transferred to polyvinylidene fluoride membranes. Membranes were blocked with 5% bovine serum albumin (BSA) (Sigma-Aldrich, Sydney, NSW, Australia) in Tris-buffer with 0.1% Tween-20 (TBS-T) for 1 h, and then incubated with mouse anti-secretory glycoprotein (ECM1) monoclonal antibody (MAb) (1:500 dilution, Abcam, USA), rabbit anti-MAST4 polyclonal antibody (PAb) (1:1000 dilution, Abcam, USA) and rabbit anti-filaggrin PAb (1:1000 dilution, Abcam, USA) at 4°C overnight (o/n), followed by incubation in horseradish peroxidase (HRP)-conjugated goat anti-rabbit or goat anti-mouse secondary antibodies (1:2000 dilution). Immunoreactive bands were detected using enhanced chemiluminescence (ECL) substrate (Pierce Chemical Co, Rockford, USA), and imaged using the ImageQuant LAS4000 system (GE Health care, USA). To confirm equal loading of protein lysates, membranes were stripped (Restore Western Blot Stripping Buffer, Pierce) and re-probed using housekeeping antibodies GAPDH (MAb: EDM Millipore) or β-tubulin (MAb: Sigma-Aldrich Pty Ltd, Australia), then processed as above. Images were processed in Adobe Photoshop.

### Immunohistochemistry

Standard immunoperoxidase procedures were used to visualize MAST4 expression using our published method [16]. Briefly, paraffin sections including BC tissues and normal breast tissues were deparaffinised in xylene, followed by a graded series of alcohols (100%, 95%, and 75%) and re-hydrated in water followed by Tris-buffered Saline (TBS) (pH 7.5). Slides were subsequently immersed in boiling 0.1 M citrate buffer (pH 6.0) for 30 min to enhance antigen retrieval, treated with 3% hydrogen peroxide and then incubated with primary rabbit anti-MAST4 PAb (1:100 dilution) o/n at 4°C. After washing with TBS, slides were incubated with goat anti-rabbit IgG (Dako, North Sydney NSW, Australia) secondary antibody (1:100 dilution) for 45 min at room temperature. Sections were finally developed with 3,3’ diaminobenzidine (DAB) substrate solution (Sigma-Aldrich, Pty Ltd, Castle Hills, NSW, Australia) as a chromogen, then counterstained with hematoxylin and blued with Scotts Bluing solution. Control slides were treated in an identical manner, and stained with an isotype matched non-specific immuno-globulin as a negative control. MDA-MB-231 cell line was used as positive control.

### Assessment of immunostaining

Staining intensity (0–3) was assessed using light microscopy (Leica microscope, Germany) at a x 40 objective as—(negative), + (weak), ++ (moderate), and +++ (strong) using our previously published method [16]. Evaluation of tissue staining was done, independently, by two experienced observers (JB and YL). All specimens were scored blind and an average of grades was taken. If discordant results were obtained, differences were resolved by joint review and consultation with a third observer, experienced in immunohistochemical pathology.

## Results and Discussion

### Proteomic discovery of circulating urinary markers in human BC

Label-free LC-MS/MS quantification was used to characterize the differential expression of urinary proteins in various human BC stages. The urine samples were analysed from patients with DCIS, IBC, MBC, BBD and normal healthy control subjects (ANOVA *p*< 0.05; q<0.02). A reverse database was also searched to determine protein level FDR. Using Progenesis software to compare protein expression between all the samples, we identified a total of 166 proteins with 1% FDR (for protein identification, determined by searching a reverse database).

**Fig 1:**
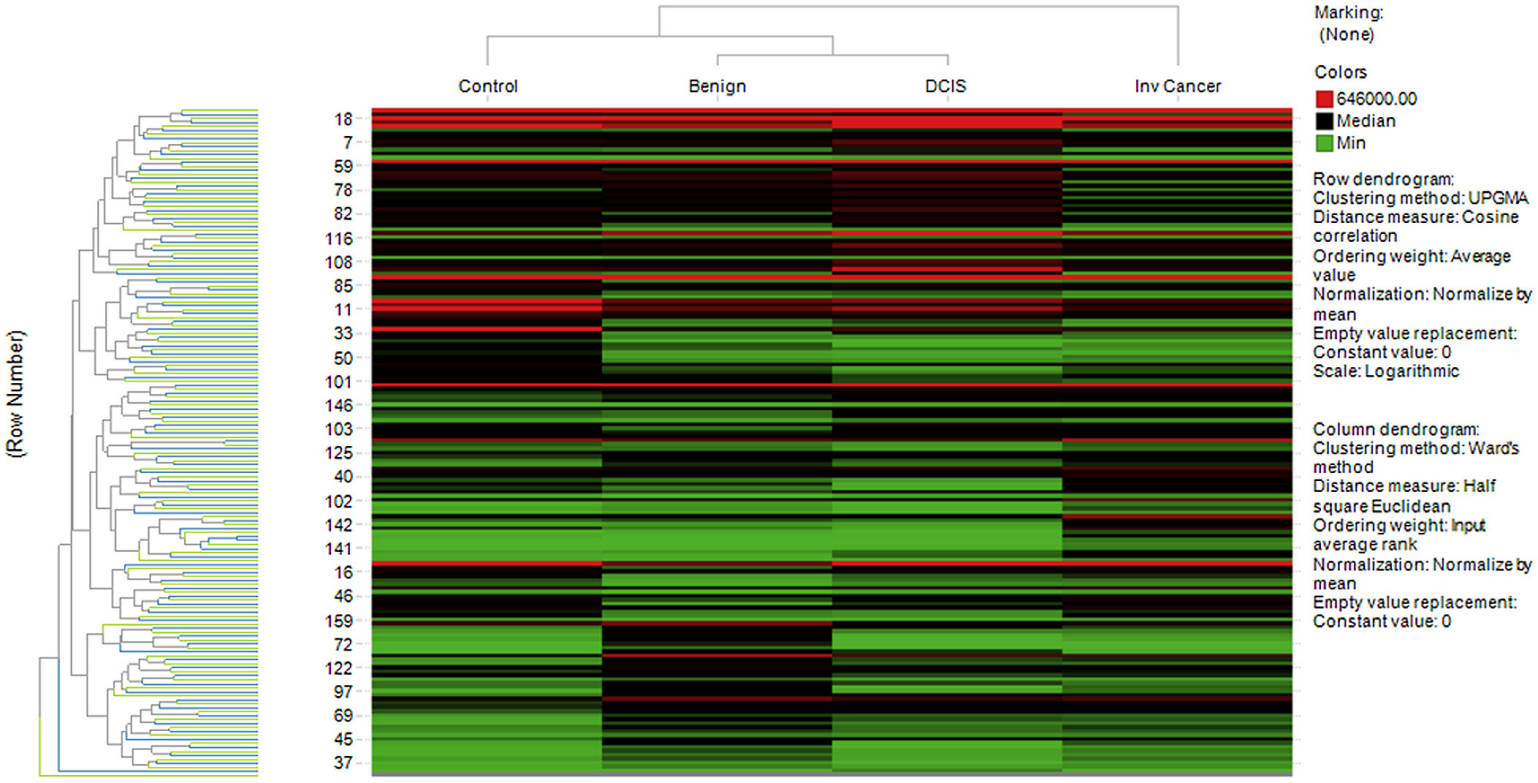
Heat map analysis of urine proteins from BC patients and conrol subjects. Heat map and dendogram of the raw data of urinary proteins identified up or down expression of urine proteins from different stages of BC patients and normal health control subjects.

Using the raw urine data and Tibco-spotfire software Inc. 2014, a biological heat map of clusters from different stages of BC patients and normal health control subjects was produced. The representative data are shown in Fig 1. This analysis demonstrates datasets as clustered patterns which show an overview of the distribution of urine proteins represented according to their expression.

The data obtained with Progenesis LC-MS analysis was then applied to calculate the fold change (FC) as a normalised ratio for disease compared to healthy control subjects. This statistical analysis revealed 59 significant urinary proteins in BC with >3-fold change relative to the normal healthy control subjects. These protein profiles are all recorded in Table 2 and S1–S3 Tables. A review of the literature demonstrates that the 59 significant proteins changing in abundance have not been detected in human urine in either BC or BBD. Several of these proteins identified were previously reported in blood, tissue and human BC cell lines (associated references are shown in Table 2 and S1–S3 Tables), supporting that these proteins detected are associated with BC. Therefore, in this study we decided to focus on the unreported proteins changing in abundance, and their biological significance in BC. In addition, several proteins typically associated with plasma (CO3, KV101, ALBU, A1AG1, FETUA, LAC2, TTHY, A1BG, CO6A1, FIBA, CERU and HAPT) which are known to be excreted in urine [17] were also detected. These proteins are shown in the Table 2 and S2 and S3 Tables with protein spots marked *. Several of plasma associated proteins (except LAC2 and FIBA) were also found to be associated with BC, further substantiating our findings.

**Table 2:** A list of urinary proteins identified by LC-MS/MS, uniquely associated with specific stages of breast cancer (DCIS, IBC and MBC).

HumanAccess. ID	Uni-Prot ID	Protein Description	Pep. ID	Score	Fold	SL	BC Ref.	Non BC Ref.
		***DCIS***						
***CO3***	**P01024**	Complement C3 * ^Ř^	2	92	3.9	S	[18–25]	
***CYTA***	**P01040**	Cystatin-A ^Ř^	4	227	3.7	C	[26]	[27,28]
***KV101***	**P01593**	Ig kappa chain V-I region AG * ^Ř^	4	385	5.3	MF	[29]	
***LRC36***	**Q1X8D7**	Leucine-rich repeat-contain protein 36 ^Đ^	2	73	3.7	CM		
***MAST4***	**O15021**	Microtubule-associated serine/ threonine-protein kinase 4	3	73	4.2	C		[30,31]
***ALBU***	**P02768**	Serum albumin * ^Ř^	44	1974	4.1	S	[32,33]	
***CI131***	**Q5VYM1**	Uncharacterized protein C9orf131	3	93	4.4	U		
		***IBC***						
***ANXA1***	**P04083**	Annexin A1 ^Ř^	6	334	3.0	C, PM	[34–37]	
***DYH8***	**Q96JB1**	Dynein heavy chain 8, axonemal	3	79	3.4	C		
***HBA***	**P69905**	Haemoglobin subunit alpha ^Đ^	3	89	3.1	C		[38,39]
***IGHG2***	**P01859**	Ig gamma-2 chain C region ^Ř^	7	250	3.2	S	[22]	
***ITIH4***	**Q14624**	Inter-alpha-trypsin inhibitor heavy chain H4 ^Ř^	10	523	14.0	S	[19,40]	[41,42]
***TRFL***	**P02788**	Lacto transferrin ^Ř^	2	100	17.6	S	[43]	[44]
***NGAL***	**P80188**	Neutrophil gelatinase-assoc. lipocalin ^Ř^	2	93	3.3	S	[45,46]	
***PEPA***	**P0DJD8**	Pepsin A ^Đ^	8	362	12.8	S	PEPC [47]	
***VTNC***	**P04004**	Vitronectin ^Ř^	2	58	4.0	S	[33,48,49]	[50]
		***MBC***						
***AGRIN***	**O00468**	Agrin ^Đ^			4.5	S, CM		[51]
***A1AG1***	**P02763**	Alpha-1-acid glycoprotein 1*^Ř^	4	182	4.4	S	[52]	
***FETUA***	**P02765**	Alpha-2-HS-glycoprotein * ^Ř^		103	3.5	S	[53,54]	
***APOA4***	**P06727**	Apolipoprotein A-IV ^Ř^	2		3.6	S	[55]	
***CAH1***	**P00915**	Carbonic anhydrase 1^Ř^	5	276	3.6	Mit	[32]	
***SULF2***	**Q8IWU5**	Extracellular sulfatase Sulf-2 ^Ř^	3	238	3.2	CS	[56,57]	
***NEGR1 (+2)***	**Q7Z3B1**	Neuronal growth regulator 1 ^Đ^	7	184	4.2	CM		[58]
***SPRL1***	**Q14515**	SPARC-like protein 1 ^Ř^	3	100	3.4	S	[59]	[60–64]

**Notes: Accession ID**, Human Accession identification;**Uni-Prot ID**, Protein identification based on the Protein knowledge base UniProtKB/Swiss-Prot ID (http://www.uniprot.org);**Pep ID**, Assigned Peptides Identified;**Score**, Mascot score;**SL**, Sub-cellular location as annotated in UniProtKB. **Fold**: Fold change for BC samples normalized against the healthy control. The proteins of interest showing biological significance are underlined. For ease of navigation, all proteins reported in the literature associated with BC are marked ^Ŕ^ or with other disease marked ^Đ^. Plasma Proteins reported in Normal Urine*[17].

Abbreviations: C, Cytoplasm; CM, Cell membrane; CS, Cell surface; MF, Membrane fraction; Mit, Mitochondrion; PM, Plasma membrane; S, Secreted; U, Unknown.

### Classification of identified urine proteins in BC

**Fig 2:**
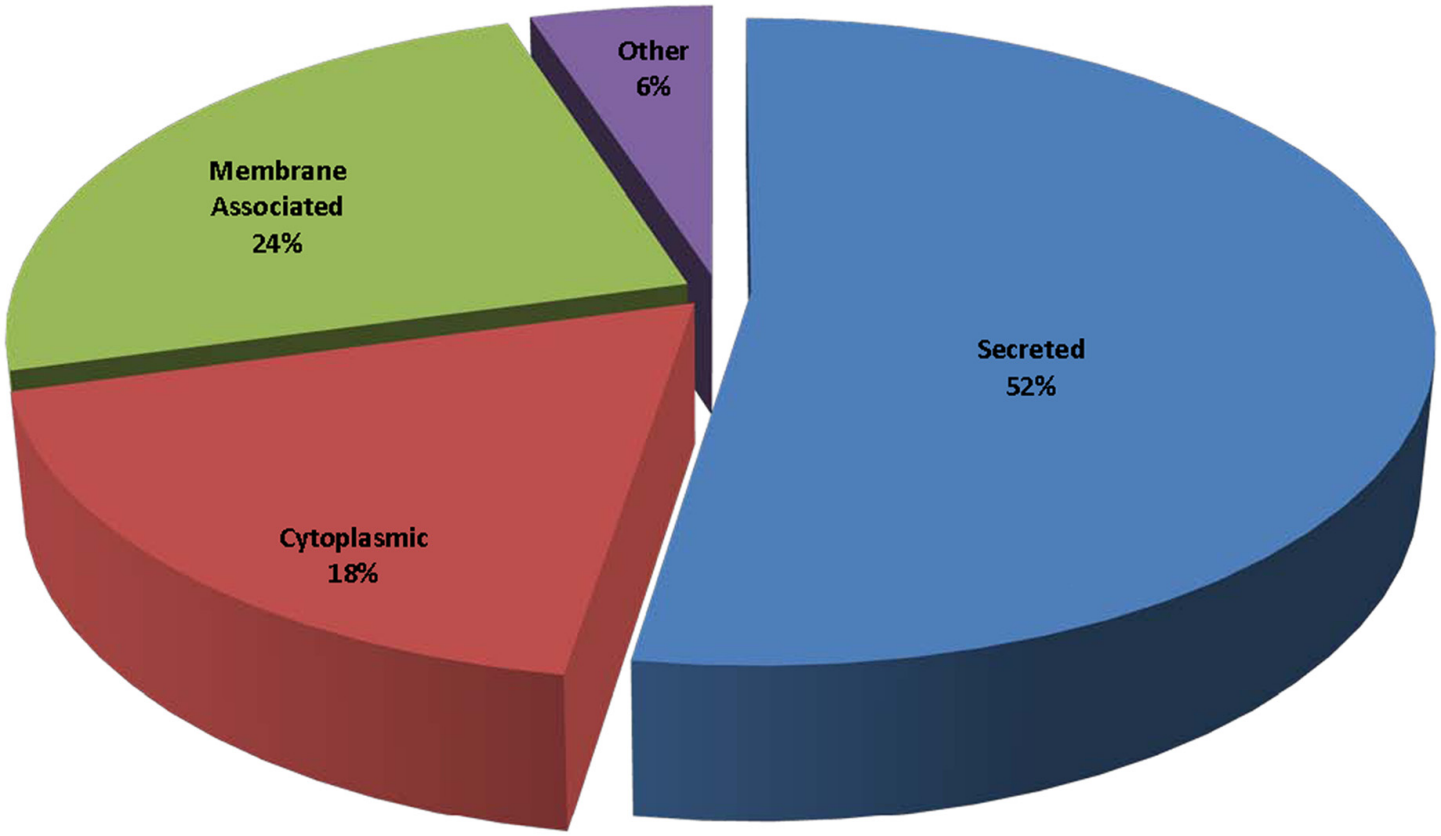
The sub-cellular locations of the 59 significant BC urinary proteins (*p*<0.05, >3-fold). Protein locations illustrate that 52% are secreted, 24% membrane-associated, 18% cytoplasmic, and 6% are either nuclear, mitochondrial, cell organelle or unknown sub-cellular origin.

The 59 significant urinary proteins (*p*<0.05, >3-fold) identified (see Table 2 and S1–S3 Tables) were classified according to their subcellular locations based on the Uni-Prot entry information available. Protein locations shown in Fig 2 demonstrate that 52% (32) of the proteins are secreted, 18% (11) cytoplasmic, 24% (15) membrane-associated, and 6% (4) grouped as others consisted of nuclear, mitochondrial, cell organelle or unknown sub-cellular origin. The majority of significant BC related proteins detected are secreted and membrane associated in nature, either tumor or host in origin but associated with the presence of disease.

### Urine protein distribution in BC patients

To investigate novel urine biomarkers in BC, we wanted to identify proteins which are both increasing and decreasing in abundance, and associated with BC prognosis. Our proteomics screening data provided a list of signature proteins for BC (Table 2, and S1 Table) and benign disease (S3 Table). Firstly, this signature list highlights 37 unique circulating proteins which were found to be expressed only in specific stages of BC and not across all the urine samples. These BC profiles included 24 up-regulated proteins (Table 2) and 12 down regulated proteins (S1 Table). Additionally, 23 proteins were identified which appeared across the different BC urine samples, some of which displayed similar patterns of protein expression in DCIS and IBC (S2 Table).

Our findings in the current study indicate that several significant urinary proteins in the BC samples had a strong relationship with BC stage and are potentially promising BC markers. Our profile lists contained several makers which have already been investigated and are known to be associated with BC (see S2 Table). Therefore, the aim of this study was to highlight the novel additional proteins which are not yet reported. We found 3 abundant proteins were associated with pre-invasive BC (i.e. DCIS patients) including Leucine-rich repeat-containing protein 36 (LRC36), Microtubule-associated serine/threonine-protein kinase 4 (MAST4) and a novel uncharacterised protein C9orf131 (CI131) (Table 2). Also in the DCIS samples, Secretogranin-1 was found to be decreased (S1 Table).

Invasive BC is a cancer stage that invades outside the basement membrane of the lobule or duct into the breast tissue, and can then spread to lymph nodes and distant organs. Finding markers to detect the cancer before it spreads would prevent life threatening metastatic disease. Several of the proteins which we detected have been reported as markers of BC and include ANXA1, Vitronectin, Lacto transferrin, ITIH4 and NGAL (see Table 2). In our findings, the six unreported potential markers of early IBC are Dynein heavy chain 8 (DYH8), Haemoglobin subunit alpha (HBA) and Pepsin A (PEPA >10 FC) (Table 2), along with uncharacterized protein C4orf14 (CD014, >200-fold), filaggrin (>30-fold) and Multimerin-2 (down >40-fold in DCIS, S2 Table), which are markedly elevated in the IBC samples. Protein DYH8 was previously detected in serum of normal health subjects [65]. Although HBA was reported as a potential serum biomarker in ovarian [39] and colon cancer [38], only hemoglobin subunit β was reported to be elevated in the BC patients [19]. Notably, Desmoglein-1, Kallikrein-1, Keratin, type II cytoskeleton 2 epidermal (K22E) and Poliovirus receptor (PVR) were all proteins significantly down regulated in IBC (S1 Table). Desmoglein-1 was reported as a prognostic marker in anal carcinoma [66]. However, only Desmoglein-3 was found to be associated with BC cells [67]. Kallikrein-1 has not been assigned for a specific biological function, even though Kallikrein-2 and 3 are serum and tissue markers for diagnosis and monitoring of prostate cancer and BC [68,69]. Poliovirus receptor (also called CD155/PVR) which was down regulated is known to have a key role in motility during cancer cell invasion, migration [70] and cell adhesion in BC cell lines [71].

MBC is a stage of the cancer which has spread to distant sites within the body. A detection pattern for MBC could provide the opportunity for early therapeutic intervention. Our analysis results provide a list of proteins with numerous proteins already linked to BC including A1AG1, FETUA, APOA4, CAH1, SULF2 and SPRL1 (see Table 2). In MBC, the novel proteins detected include AGRIN, and Neural growth regulator 1 (NEGR1) (Table 2), as well as Fibrinogen alpha chain (FIBA) and Keratin type 1 cytoskeleton 10 (KIC10) (S2 Table) which were exclusively elevated in the MBC samples. Only nerve growth factor 1 (NEGF1) was previously reported in relation to the survival and proliferation of BC cells [72,73]. Additionally, in this group of patients, Vasorin and Vitelline membrane outer layer protein 1 homolog (VMO1) were both down regulated (S1 Table).

Analysis of the differential expression patterns of urine samples across different stages of BC highlights potential protein markers that can identify some similarities or difference between DCIS and IBC. DCIS is a non-invasive process which can progress to IBC. If a biomarker or a panel of biomarkers could be identified in DCIS stage, an early action taken may prevent IBC occurrence. Therefore, the identified potential BC markers have clinical significance.

The 23 proteins listed in S2 Table include 3 potential progression markers of BC: immune response proteins immunoglobulin (Ig) kappa chain V-I region WEA (KV118), lambda -2 chain C region (LAC2) and ECM1. Protein ECM1 was elevated in BC (S2 Table). ECM1 is a secreted glycoprotein, previously reported to be associated with BC metastatic bone homing [74] and plays an important role in BC progression [75]. High levels are detected in aggressive tumorigenic cancer cell lines MDA435 [76], in ductal breast carcinomas [77] and its expression is also correlated with poor prognosis [78] and metastatic potential in cancer [79]. In the current study, this protein was detected in IBC urine samples and validated in primary and metastatic BC cell lines, further confirmed its link with BC patients. This urine marker ECM1 demonstrates the potential for BC diagnosis and monitoring.

### Urine protein distribution in benign disease patients

Although some benign breast diseases are associated with increased risk of subsequent BC, benign breast condition is generally considered as a noncancerous disorder. In such cases, markers at this stage are important for early detection in high risk individuals and understanding of disease progression. In this study, we also identified a list of unreported proteins of interest in association with BBD (S2 and S3 Tables). The 6 up-regulated proteins uniquely found only in BBD include FAM184A, transcription factor E2F8 and Cadherin-1 along with Ig-response proteins kappa chain V-III region VG (KV309, >1000-FC), lambda like polypeptide 5 (IGLL5) (S2 Table), and Ig kappa chain V-I region BAN (KV122) (S3 Table). Several down regulated proteins were also identified in the benign breast patients (S3 Table). The most significant benign breast protein identified was Nucleobindin-1, which has a 200-fold change (S2 Table). Nucleobindin-1 is a major intracellular calcium-binding protein previously detected in colorectal cancer cells after treatment with anti-tumor compounds [80].

### Ingenuity analysis of interaction networks of human urine proteins in BC patients

**Fig 3:**
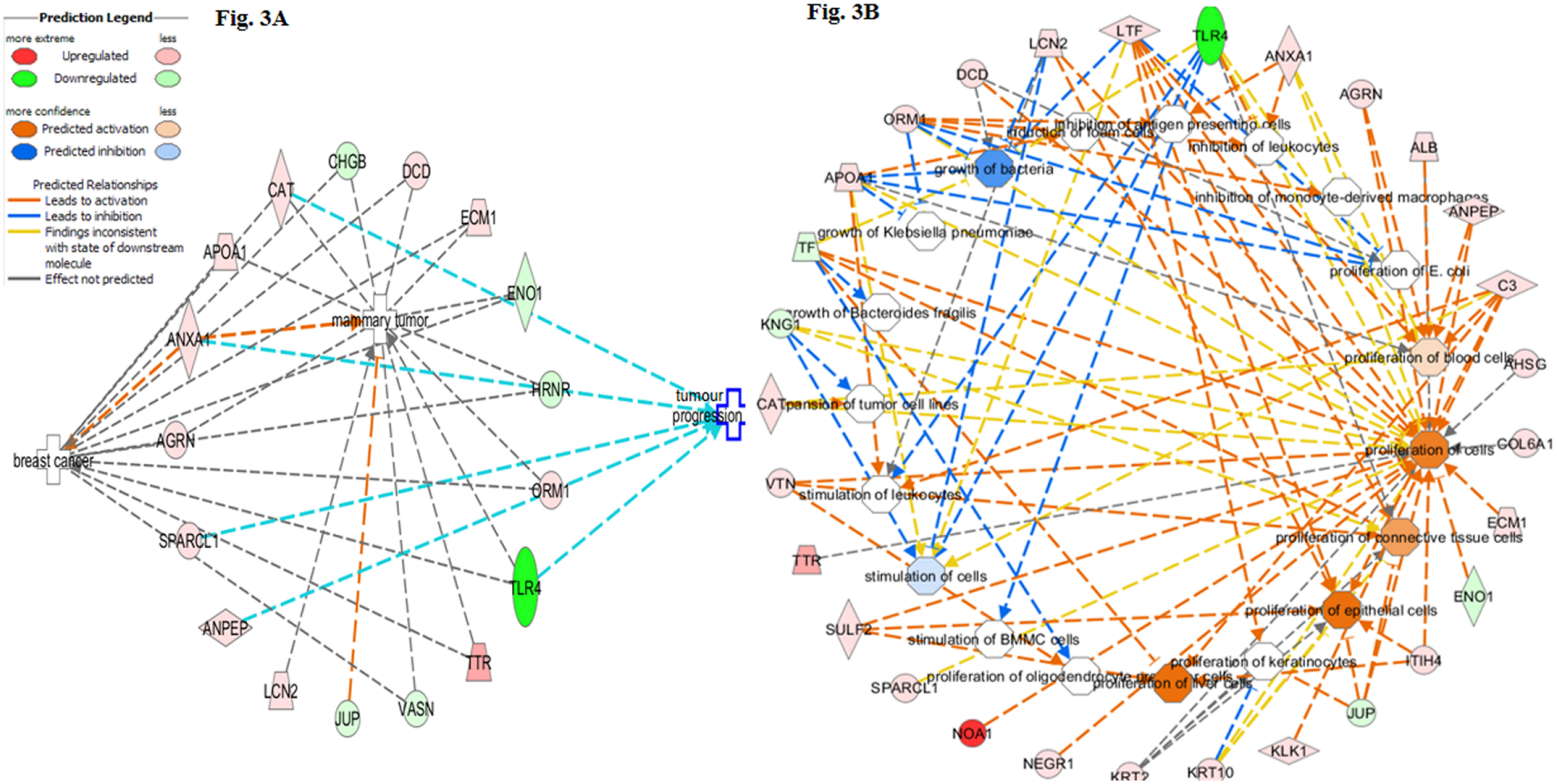
Ingenuity cell growth and proliferation analysis of the urine proteins detected in BC. **A**. The cell growth and proliferation analysis showing the urine proteins detected in BC patients are associated with tumor growth and progression. **B**. The results demonstrating that the strong biological involvement and the direct effect of these identified proteins have their roles in inhibition and proliferation of various cells in the body.

**Fig 4:**
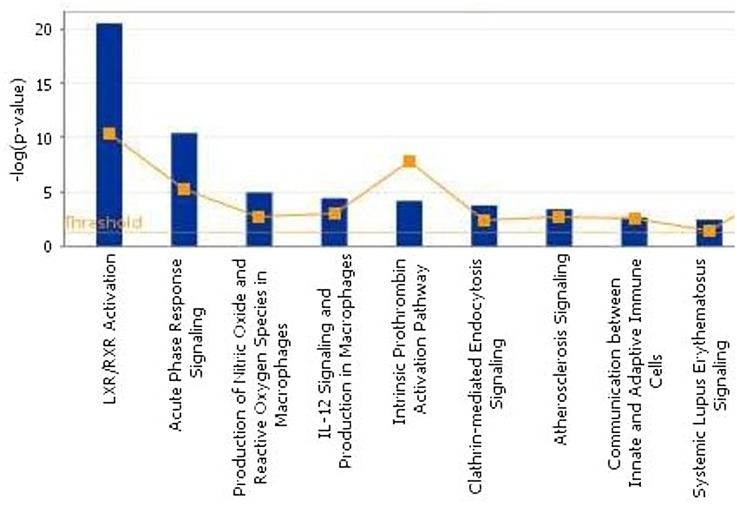
Ingenuity pathway analysis showing the top enriched canonical pathways. The enrichment of canonical pathways in the significant up-regulated urine proteins associated with BC.

To identify the major biologic pathways of BC urine involved, Ingenuity Pathway Analysis (IPA) Software (IPA; Ingenuity^®^ Systems, www.ingenuity.com Release date: 05-02-2013, Ingenuity Systems, Redwood City, CA, USA) was used for canonical pathway enrichment analysis and the derivation of mechanistic networks. The lists of proteins were uploaded directly into IPA for analysis, and functional pathways or networks with the highest confidence scores were then determined. Cell growth and proliferation analysis are shown in Fig 3. The proteins detected from Table 2 and S1 and S2 Tables are found to be associated with tumor growth and progression (Fig 3A), suggesting that these proteins are involved in the inhibition or proliferation of various cells in BC patients (Fig 3B). The enriched pathways associated with BC urine are shown in S1 Fig while the best scored networks were selected are shown in Fig 4. Highly interconnected networks are likely to represent significant biological functions associated with BC progression.

Proteomics results from this study demonstrate that the proteins detected in BC urine are involved in the LXR/RXR activation and acute-phase response pathways, which are active during inflammation and/or as a contribution of the immune response to cancer. Other pathways also enriched include production of nitric oxide and reactive oxygen species (ROS) in macrophages and IL12 signaling and production in macrophages (Fig 4). IPA highlighted cholesterol metabolism as significant in our BC samples. Cholesterol is an essential structural component of the cell membrane and proliferating cells. Cancer cells are believed to have increased requirements for cholesterol. Cancer cells can increase lipid biosynthesis and uptake cholesterol from the bloodstream [81]. It seems that LDL-cholesterol enriched systemic environment promotes BC progression by activating key signaling pathways and modulating cell behaviour. LDL-cholesterol signaling was shown to induce BC proliferation and invasion [82].

These canonical pathway results show that multiple pathways and networks are involved in the systemic response to BC and that intrinsic and endocytosis signaling pathways play a role, along with communication between the innate and the adaptive immune system. Increasing evidence indicates that the immune response plays an important role in BC disease [83,84]. Therefore, these immune response proteins in urine have potential to be used as BC biomarkers for diagnosis and monitoring. The interaction between these identified pathways in patients’ urine and BC disease is complex and biologically significant. More efforts will be put to study the roles and functions of these signaling pathways in BC in the future.

### Validation of the identified potential urine markers in BC cell lines

**Fig 5:**
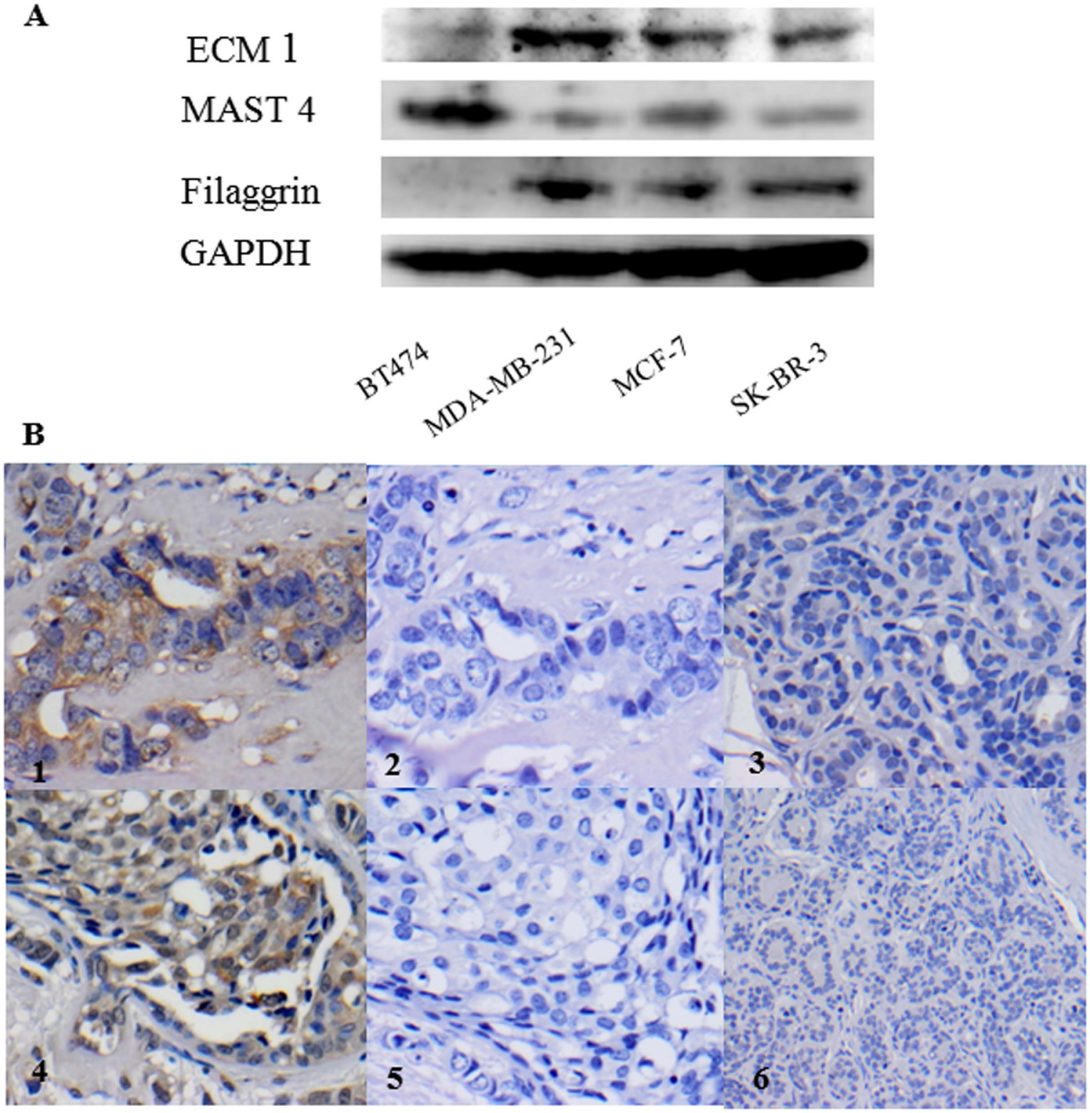
Validation of identified potential urine proteins ECM1, MAST4 and filaggrin from BC patients in human BC cell lines and protein MAST 4 in primary BC tissues. **A.** High level of MAST4 was found in the primary BC cell line (BT474) and medium levels of MAST4 were found in the metastatic BC cell lines (MDA-MB231, MCF-7 and SKBR-3). High levels of ECM1 and filaggrin were found in the metastatic BC cell lines (MDA-MB231, MCF-7 and SKBR-3) while low level of ECM1was seen in the primary BC cell line (BT474) and no filaggrin expression was detected in the primary BC cell line (BT474). *GAPDH* was used as a loading control. **B.** Illustration of the positive expression of MAST4 in BC using immunohistochemistry. Moderate cytoplasmic expression of MAST4 (++) were seen in the primary IBC (B1) and DCIS (B4) tumours (n = 5 for each stage of BC); there was no expression of MAST4 in the negative controls for either IBC (B2) or DCIS (B5). No staining was seen for MAST4 in normal breast tissues (B3 and B6), (n = 5). Magnifications x 400 in B1, B2, B4 and B5; magnifications x 200 in B3; magnifications x 100 in B6. Brown indicates positive staining and blue indicates nuclei. All results were from 3 independent experiments (n = 3).

To find an association of identified potential urine protein with human BC, one existing marker-ECM1 and another two selected novel protein markers MAST4, and filaggrin were evaluated in human primary BC cell line (BT474) and metastatic BC cell lines (MDA-MB-231, MCF-7 and SK-BR-3) by Western blotting. As shown in Fig 5A, ECM1 and MAST4 were positive in all 4 BC cell lines and filaggrin was positive in the 3 metastatic BC cell lines, suggesting the identified potential urine markers from BC patients, are closely associated with human BC.

### Preliminary validation of identified potential marker MAST4 in human primary BC tissues

To further investigate the clinical significance of our findings, we conducted immuno-histochemistry (IHC) for preliminary validation of one novel protein marker MAST4 using a small number of representative human primary BC tissue samples, including DCIS and IBC patients and normal breast tissues. Our results indicate that MAST4 was positive in 80% (4/5) of IBC and in 60% (3/5) of DCIS, respectively and no positive staining was seen in normal breast tissues (5/5). The typical staining results are shown in Fig 5B. These findings further strengthen the link of the identified potential urine marker with BC disease. Due to the limited number of patients’ tissue samples, a lager sample size is required in the following study.

### Preliminary validation of potential mark MAST4 in individual human BC urine samples

**Fig 6:**
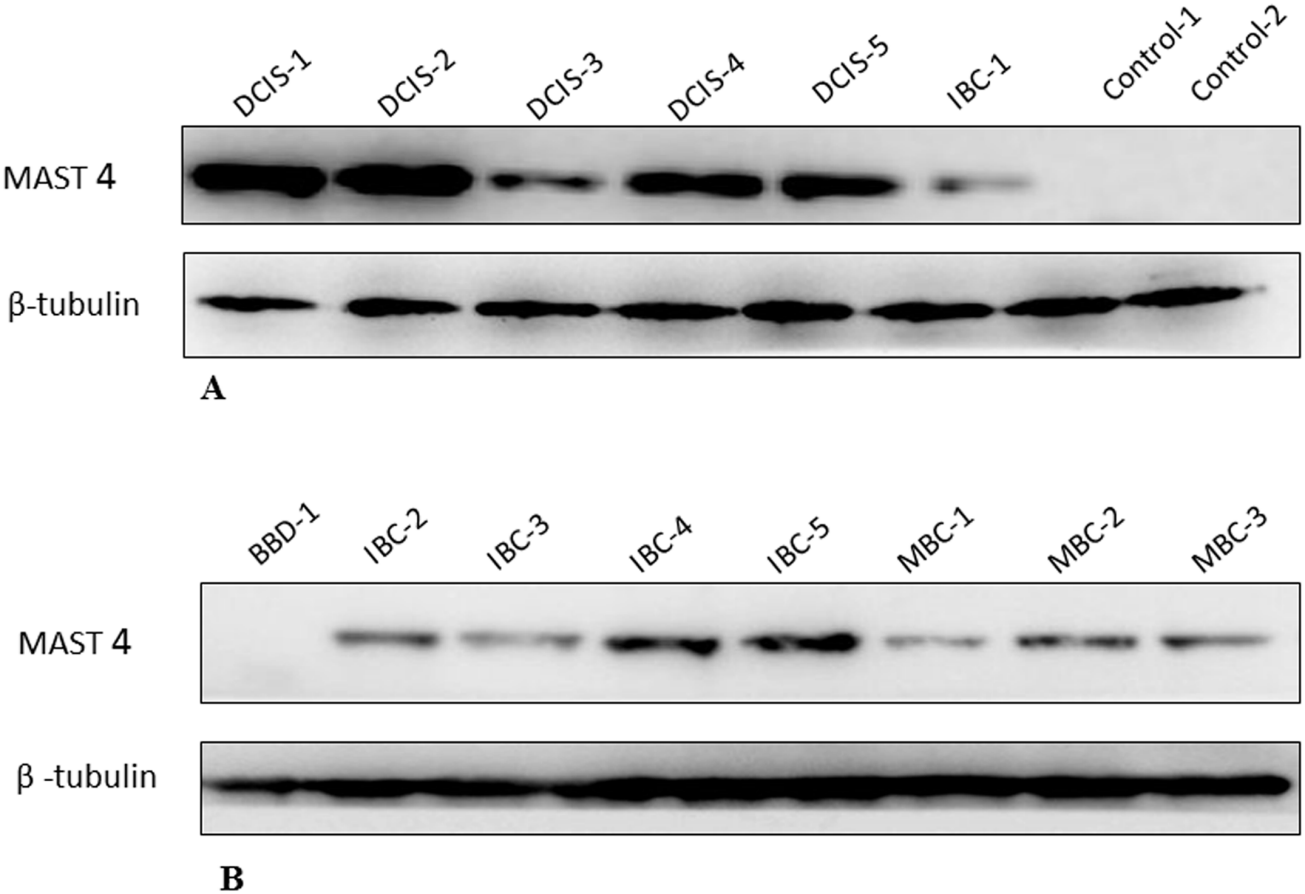
Expression of MAST4 in the individual urine samples from BC patients and controls. Western blotting was performed on the remaining urine samples from BC patients and control subjects to confirm the expression of MAST4. **A.** Overexpression of MAST4 was shown in the individual DCIS urine samples (DCIS 1–5), weak expression in IBC1 urine sample and no expression in control urine samples. **B**. There was no MAST4 expression found in the BBD urine sample but medium expression in IBC (IBC 2–5) and MBC urine samples (MBC 1–3). ß-tubulin was chosen as a loading control.

In order to confirm MAST4 overexpression in the individual DCIS patients, the remaining BC urine samples available were re-examined using Western blotting. Our results clearly indicate that high levels of MAST4 expression were found in the individual DCIS urine samples and low levels in IBC and MBC urine samples, while no expression was seen in the samples from BBD patients and normal health control subjects (see Fig 6), further confirming that a strong link exists between MAST4 protein and the DCIS urine samples identified with the LC-MS/MS.

MAST4 is a protein-coding gene. The roles and functions of this protein in cancers have not been reported so far. Differential expression of MAST4 was observed between the four different cell lines. High level of MAST4 expression was found in BT474 cell line which is an aggressive luminal B subtype, suggesting this protein could be used as a therapeutic target of interest for future studies of endocrine resistance. Though proteomics screening detected MAST4 as a significant increased marker in DCIS, our results from BC cell lines and human BC tissues suggest that MAST4 may be also involved in BC progression, which is also in line with the positive expression in the individual urine samples from IBC and MBC patients. A preliminary analysis of publically available mRNA expression profiling data (Kaplan-Meier Plotter, an online survival analysis tool [85] http://kmplot.com/analysis/index.php?p=service&cancer=breast), demonstrated that MAST4 expression is highly significantly correlated with survival in a cohort of over 3,000 BC patients. Therefore, we plan to further investigate the MAST4 expression in a large independent cohort of BC patients and study its role in BC metastasis.

## Conclusions

Identification of new biomarkers in early and advanced BC is important in the prevention and monitoring of disease progression and is a recently developed research area. MS is a very powerful technique for comprehensive analysis of proteins. Collection of urine from patients is relatively easy and non-invasive, therefore making it an ideal candidate to sample for clinical management of patients and to search for biomarkers.

Within BC publications, most studies have used MS technologies to analyse urine metabolomic biomarkers. In this study, LC-MS/MS analysis was performed on urine samples from BC patients, benign patients and healthy control subjects. Therefore the results of this comparative discovery study provide a panel of novel significantly altered urinary proteins that are abundant in pre-invasive and invasive BC, but have not yet been previously detected in urine or other biological samples. These BC proteins identified provide further insight into the complex signaling pathway interactions occurring during the progression of BC. Our study indicates that the majority of the abundant BC urinary proteins detected are secreted proteins. The list of novel up-regulated proteins detected (Table 2), provides information which could be used to create a panel of targets which could form part of urine screening “dipstick” test for the detection of non-invasive and invasive BC.

Breast cancer cell lines are preclinical models that represent different breast tumor subtypes. To link the potential of urine markers identified with BC disease, we validated one existing marker and two novel biomarkers in human BC cell lines by Western blot analysis. We demonstrated significantly elevated expression of three interesting markers-ECM1, MAST4 and filaggrin in a panel of human BC cell lines and one marker MAST4 in a small group of clinical BC tissue (Fig 5) and urine (Fig 6) samples, indicating their promise for further investigation.

Urinary proteins can potentially provide a preliminary indication of the presence of BC during screening and could assist with direct examination and pathology testing for final diagnosis. The development of a non-invasive test of BC risk has been a major goal for more than 20 years. In the current study, we present potential protein biomarkers that are related to BC stage that can be used for early diagnosis and monitoring cancer progression in urine. These novel protein markers in urine require to be further evaluated in BC tissues and an independent group of BC urine samples to test their specificity and sensitivity for BC early diagnosis and lead to potential applications in cancer surveillance and prevention.

## Supporting Information

S1 Fig In silico identification of interactive networks using BC urinary proteins analysed with LC-MS/MS. (TIFF)

S1 Table A list of proteins with decreased expression in urine, uniquely associated with certain stages of breast cancer (DCIS, IBC and MBC). (DOCX)

S2 Table A list of differentially expressed urinary proteins in breast cancer and benign breast disease. (DOCX)

S3 Table A list of urine proteins up-and-down regulated in benign breast disease. (DOCX)

## References

[1] Lakhani SR, Ellis IO, Schnitt SJ, Tan PH, van de Vijver MJ, editors (2012) WHO Classification of Tumours of the Breast, Fourth Edition Lyon: IARC WHO Classification of Tumours.

[2] Yates JR, Ruse CI, Nakorchevsky A (2009) Proteomics by mass spectrometry: approaches, advances, and applications. Annual Review of Biomedical Engineering 11: 49–79. 10.1146/annurev-bioeng-061008-124934 19400705

[3] Pan S, Chen R, Aebersold R, Brentnall TA (2011) Mass spectrometry based glycoproteomics—from a proteomics perspective. Molecular & Cellular Proteomics 10: R110.003251. 10.1074/mcp.R110.003251 PMC3013464 20736408

[4] Adachi J, Kumar C, Zhang Y, Olsen JV, Mann M (2006) The human urinary proteome contains more than 1500 proteins, including a large proportion of membrane proteins. Genome Biology 7: R80 16948836 10.1186/gb-2006-7-9-r80 PMC1794545

[5] Thongboonkerd V, McLeish KR, Arthur JM, Klein JB (2002) Proteomic analysis of normal human urinary proteins isolated by acetone precipitation or ultracentrifugation. Kidney International 62: 1461–1469. 12234320 10.1111/j.1523-1755.2002.kid565.x

[6] Husi H, Stephens N, Cronshaw A, MacDonald A, Gallagher I, et al (2011) Proteomic analysis of urinary upper gastrointestinal cancer markers. Proteomics Clinical Applications 5: 289–299. 10.1002/prca.201000107 21538913

[7] Lei T, Zhao X, Jin S, Meng Q, Zhou H, et al (2012) Discovery of Potential Bladder Cancer Biomarkers by Comparative Urine Proteomics and Analysis Clinical genitourinary cancer. 10.1016/j.clgc.2012.06.003 22982111

[8] Hassanein M, Callison JC, Callaway-Lane C, Aldrich MC, Grogan EL, et al (2012) The state of molecular biomarkers for the early detection of lung cancer. Cancer prevention research (Philadelphia, Pa) 5: 992–1006. 10.1158/1940-6207.CAPR-11-0441 PMC3723112 22689914

[9] Beretov J, Wasinger VC, Graham PH, Millar EK, Kearsley JH, et al (2014) Proteomics for breast cancer urine biomarkers. Adv Clin Chem 63: 123–167. 24783353 10.1016/b978-0-12-800094-6.00004-2

[10] Linden M, Lind SB, Mayrhofer C, Segersten U, Wester K, et al (2012) Proteomic analysis of urinary biomarker candidates for nonmuscle invasive bladder cancer. Proteomics 12: 135–144. 10.1002/pmic.201000810 22065568

[11] Zoidakis J, Makridakis M, Zerefos PG, Bitsika V, Esteban S, et al (2012) Profilin 1 is a potential biomarker for bladder cancer aggressiveness. Molecular & cellular proteomics: MCP 11: M111 009449. 10.1074/mcp.M111.009449 PMC3322560 22159600

[12] Beretov J, Wasinger VC, Schwartz P, Graham PH, Li Y (2014) A standardized and reproducible urine preparation protocol for cancer biomarkers discovery. Biomark Cancer 6: 21–27. 10.4137/BIC.S17991 25452700 PMC4219630

[13] Karp NA, McCormick PS, Russell MR, Lilley KS (2007) Experimental and statistical considerations to avoid false conclusions in proteomics studies using differential in-gel electrophoresis. Mol Cell Proteomics 6: 1354–1364. 17513293 10.1074/mcp.M600274-MCP200

[14] Storey JD, Tibshirani R (2003) Statistical significance for genomewide studies. Proc Natl Acad Sci U S A 100: 9440–9445. 12883005 10.1073/pnas.1530509100 PMC170937

[15] Chang L, Graham PH, Hao J, Ni J, Bucci J, et al (2013) Acquisition of epithelial-mesenchymal transition and cancer stem cell phenotypes is associated with activation of the PI3K/Akt/mTOR pathway in prostate cancer radioresistance. Cell Death Dis 4: e875 10.1038/cddis.2013.407 24157869 PMC3920940

[16] Hao J, Chen H, Madigan MC, Cozzi PJ, Beretov J, et al (2010) Co-expression of CD147 (EMMPRIN), CD44v3-10, MDR1 and monocarboxylate transporters is associated with prostate cancer drug resistance and progression. Br J Cancer 103: 1008–1018. 10.1038/sj.bjc.6605839 20736947 PMC2965856

[17] Candiano G, Santucci L, Petretto A, Bruschi M, Dimuccio V, et al (2010) 2D-electrophoresis and the urine proteome map: where do we stand? Journal of Proteomics 73: 829–844. 10.1016/j.jprot.2009.12.003 20004755

[18] Dowling P, Clarke C, Hennessy K, Torralbo-Lopez B, Ballot J, et al (2012) Analysis of acute-phase proteins, AHSG, C3, CLI, HP and SAA, reveals distinctive expression patterns associated with breast, colorectal and lung cancer. Int J Cancer 131: 911–923. 10.1002/ijc.26462 21953030

[19] Nasim FU, Ejaz S, Ashraf M, Asif AR, Oellerich M, et al (2012) Potential biomarkers in the sera of breast cancer patients from bahawalpur, pakistan. Biomark Cancer 4: 19–34. 10.4137/BIC.S10502 24179392 PMC3791917

[20] Zeng Z, Hincapie M, Haab BB, Hanash S, Pitteri SJ, et al (2010) The development of an integrated platform to identify breast cancer glycoproteome changes in human serum. J Chromatogr A 1217: 3307–3315. 10.1016/j.chroma.2009.09.029 19782370 PMC4142217

[21] Solassol J, Rouanet P, Lamy PJ, Allal C, Favre G, et al (2010) Serum protein signature may improve detection of ductal carcinoma in situ of the breast. Oncogene 29: 550–560. 10.1038/onc.2009.341 19855429

[22] Cho W, Jung K, Regnier FE (2010) Sialylated Lewis x antigen bearing glycoproteins in human plasma. J Proteome Res 9: 5960–5968. 10.1021/pr100747p 20858014 PMC2976037

[23] Li J, Orlandi R, White CN, Rosenzweig J, Zhao J, et al (2005) Independent validation of candidate breast cancer serum biomarkers identified by mass spectrometry. Clin Chem 51: 2229–2235. 16223889 10.1373/clinchem.2005.052878

[24] Goncalves A, Esterni B, Bertucci F, Sauvan R, Chabannon C, et al (2006) Postoperative serum proteomic profiles may predict metastatic relapse in high-risk primary breast cancer patients receiving adjuvant chemotherapy. Oncogene 25: 981–989. 16186794 10.1038/sj.onc.1209131

[25] Profumo A, Mangerini R, Rubagotti A, Romano P, Damonte G, et al (2013) Complement C3f serum levels may predict breast cancer risk in women with gross cystic disease of the breast. J Proteomics 85: 44–52. 10.1016/j.jprot.2013.04.029 23639844

[26] Parker BS, Ciocca DR, Bidwell BN, Gago FE, Fanelli MA, et al (2008) Primary tumour expression of the cysteine cathepsin inhibitor Stefin A inhibits distant metastasis in breast cancer. J Pathol 214: 337–346. 17985332 10.1002/path.2265

[27] Chang KP, Wu CC, Chen HC, Chen SJ, Peng PH, et al (2010) Identification of candidate nasopharyngeal carcinoma serum biomarkers by cancer cell secretome and tissue transcriptome analysis: potential usage of cystatin A for predicting nodal stage and poor prognosis. Proteomics 10: 2644–2660. 10.1002/pmic.200900620 20461718

[28] Leinonen T, Pirinen R, Bohm J, Johansson R, Rinne A, et al (2007) Biological and prognostic role of acid cysteine proteinase inhibitor (ACPI, cystatin A) in non-small-cell lung cancer. J Clin Pathol 60: 515–519. 16790691 10.1136/jcp.2006.038711 PMC1994551

[29] Whiteside TL, Ferrone S (2012) For breast cancer prognosis, immunoglobulin kappa chain surfaces to the top. Clin Cancer Res 18: 2417–2419. 10.1158/1078-0432.CCR-12-0566 22442060 PMC3426232

[30] Sun L, Gu S, Li X, Sun Y, Zheng D, et al (2006) [Identification of a novel human MAST4 gene, a new member of the microtubule associated serine-threonine kinase family]. Mol Biol (Mosk) 40: 808–815. 17086981

[31] Robinson DR, Kalyana-Sundaram S, Wu YM, Shankar S, Cao X, et al (2011) Functionally recurrent rearrangements of the MAST kinase and Notch gene families in breast cancer. Nat Med 17: 1646–1651. 10.1038/nm.2580 22101766 PMC3233654

[32] Somiari RI, Sullivan A, Russell S, Somiari S, Hu H, et al (2003) High-throughput proteomic analysis of human infiltrating ductal carcinoma of the breast. Proteomics 3: 1863–1873. 14625848 10.1002/pmic.200300560

[33] Kadowaki M, Sangai T, Nagashima T, Sakakibara M, Yoshitomi H, et al (2011) Identification of vitronectin as a novel serum marker for early breast cancer detection using a new proteomic approach. J Cancer Res Clin Oncol 137: 1105–1115. 10.1007/s00432-010-0974-9 21253761 PMC11827956

[34] Cao Y, Li Y, Edelweiss M, Arun B, Rosen D, et al (2008) Loss of annexin A1 expression in breast cancer progression. Appl Immunohistochem Mol Morphol 16: 530–534. 10.1097/PAI.0b013e31817432c3 18776816

[35] Wang LP, Bi J, Yao C, Xu XD, Li XX, et al (2010) Annexin A1 expression and its prognostic significance in human breast cancer. Neoplasma 57: 253–259. 20353277 10.4149/neo_2010_03_253

[36] Shen D, Chang HR, Chen Z, He J, Lonsberry V, et al (2005) Loss of annexin A1 expression in human breast cancer detected by multiple high-throughput analyses. Biochem Biophys Res Commun 326: 218–227. 15567174 10.1016/j.bbrc.2004.10.214

[37] Yom CK, Han W, Kim SW, Kim HS, Shin HC, et al (2011) Clinical significance of annexin A1 expression in breast cancer. J Breast Cancer 14: 262–268. 10.4048/jbc.2011.14.4.262 22323911 PMC3268921

[38] Rho JH, Qin S, Wang JY, Roehrl MH (2008) Proteomic expression analysis of surgical human colorectal cancer tissues: up-regulation of PSB7, PRDX1, and SRP9 and hypoxic adaptation in cancer. J Proteome Res 7: 2959–2972. 10.1021/pr8000892 18549262 PMC2693877

[39] Woong-Shick A, Sung-Pil P, Su-Mi B, Joon-Mo L, Sung-Eun N, et al (2005) Identification of hemoglobin-alpha and -beta subunits as potential serum biomarkers for the diagnosis and prognosis of ovarian cancer. Cancer Sci 96: 197–201. 15771624 10.1111/j.1349-7006.2005.00029.x PMC11158023

[40] Mohamed E, Abdul-Rahman PS, Doustjalali SR, Chen Y, Lim BK, et al (2008) Lectin-based electrophoretic analysis of the expression of the 35 kDa inter-alpha-trypsin inhibitor heavy chain H4 fragment in sera of patients with five different malignancies. Electrophoresis 29: 2645–2650. 10.1002/elps.200700828 18494030

[41] Abdullah-Soheimi SS, Lim BK, Hashim OH, Shuib AS (2010) Patients with ovarian carcinoma excrete different altered levels of urine CD59, kininogen-1 and fragments of inter-alpha-trypsin inhibitor heavy chain H4 and albumin. Proteome Sci 8: 58 10.1186/1477-5956-8-58 21083881 PMC2998473

[42] Zhang Z, Bast RC Jr, Yu Y, Li J, Sokoll LJ, et al (2004) Three biomarkers identified from serum proteomic analysis for the detection of early stage ovarian cancer. Cancer Res 64: 5882–5890. 15313933 10.1158/0008-5472.CAN-04-0746

[43] Schulz DM, Bollner C, Thomas G, Atkinson M, Esposito I, et al (2009) Identification of differentially expressed proteins in triple-negative breast carcinomas using DIGE and mass spectrometry. J Proteome Res 8: 3430–3438. 10.1021/pr900071h 19485423

[44] Deng M, Zhang W, Tang H, Ye Q, Liao Q, et al (2013) Lactotransferrin acts as a tumor suppressor in nasopharyngeal carcinoma by repressing AKT through multiple mechanisms. Oncogene 32: 4273–4283. 10.1038/onc.2012.434 23069661

[45] Wenners AS, Mehta K, Loibl S, Park H, Mueller B, et al (2012) Neutrophil gelatinase-associated lipocalin (NGAL) predicts response to neoadjuvant chemotherapy and clinical outcome in primary human breast cancer. PLoS One 7: e45826 10.1371/journal.pone.0045826 23056218 PMC3467272

[46] Provatopoulou X, Gounaris A, Kalogera E, Zagouri F, Flessas I, et al (2009) Circulating levels of matrix metalloproteinase-9 (MMP-9), neutrophil gelatinase-associated lipocalin (NGAL) and their complex MMP-9/NGAL in breast cancer disease. BMC Cancer 9: 390 10.1186/1471-2407-9-390 19889214 PMC2775750

[47] Vizoso F, Sanchez LM, Diez-Itza I, Merino AM, Lopez-Otin C (1995) Pepsinogen C is a new prognostic marker in primary breast cancer. J Clin Oncol 13: 54–61. 7799043 10.1200/JCO.1995.13.1.54

[48] Aaboe M, Offersen BV, Christensen A, Andreasen PA (2003) Vitronectin in human breast carcinomas. Biochim Biophys Acta 1638: 72–82. 12757937 10.1016/s0925-4439(03)00059-0

[49] Kim BK, Lee JW, Park PJ, Shin YS, Lee WY, et al (2009) The multiplex bead array approach to identifying serum biomarkers associated with breast cancer. Breast Cancer Res 11: R22 10.1186/bcr2247 19400944 PMC2688951

[50] Peng L, Liu J, Li YM, Huang ZL, Wang PP, et al (2013) Serum proteomics analysis and comparisons using iTRAQ in the progression of hepatitis B. Exp Ther Med 6: 1169–1176. 24223640 10.3892/etm.2013.1310 PMC3820766

[51] Klein-Scory S, Kubler S, Diehl H, Eilert-Micus C, Reinacher-Schick A, et al (2010) Immunoscreening of the extracellular proteome of colorectal cancer cells. BMC Cancer 10: 70 10.1186/1471-2407-10-70 20184735 PMC2837015

[52] Semaan SM, Wang X, Marshall AG, Sang QX (2012) Identification of Potential Glycoprotein Biomarkers in Estrogen Receptor Positive (ER+) and Negative (ER-) Human Breast Cancer Tissues by LC-LTQ/FT-ICR Mass Spectrometry. J Cancer 3: 269–284. 10.7150/jca.4592 22773931 PMC3390597

[53] Yi JK, Chang JW, Han W, Lee JW, Ko E, et al (2009) Autoantibody to tumor antigen, alpha 2-HS glycoprotein: a novel biomarker of breast cancer screening and diagnosis. Cancer Epidemiol Biomarkers Prev 18: 1357–1364. 10.1158/1055-9965.EPI-08-0696 19423516

[54] Opstal-van Winden AW, Krop EJ, Karedal MH, Gast MC, Lindh CH, et al (2011) Searching for early breast cancer biomarkers by serum protein profiling of pre-diagnostic serum; a nested case-control study. BMC Cancer 11: 381 10.1186/1471-2407-11-381 21871081 PMC3189190

[55] Custodio A, Lopez-Farre AJ, Zamorano-Leon JJ, Mateos-Caceres PJ, Macaya C, et al (2012) Changes in the expression of plasma proteins associated with thrombosis in BRCA1 mutation carriers. J Cancer Res Clin Oncol 138: 867–875. 10.1007/s00432-012-1161-y 22311183 PMC11824561

[56] Morimoto-Tomita M, Uchimura K, Bistrup A, Lum DH, Egeblad M, et al (2005) Sulf-2, a proangiogenic heparan sulfate endosulfatase, is upregulated in breast cancer. Neoplasia 7: 1001–1010. 16331886 10.1593/neo.05496 PMC1502017

[57] Khurana A, Jung-Beom D, He X, Kim SH, Busby RC, et al (2013) Matrix detachment and proteasomal inhibitors diminish Sulf-2 expression in breast cancer cell lines and mouse xenografts. Clin Exp Metastasis 30: 407–415. 10.1007/s10585-012-9546-5 23412907 PMC3619208

[58] Takita J, Chen Y, Okubo J, Sanada M, Adachi M, et al (2011) Aberrations of NEGR1 on 1p31 and MYEOV on 11q13 in neuroblastoma. Cancer Sci 102: 1645–1650. 10.1111/j.1349-7006.2011.01995.x 21624008

[59] Bellahcene A, Castronovo V (1995) Increased expression of osteonectin and osteopontin, two bone matrix proteins, in human breast cancer. Am J Pathol 146: 95–100. 7856741 PMC1870781

[60] Esposito I, Kayed H, Keleg S, Giese T, Sage EH, et al (2007) Tumor-suppressor function of SPARC-like protein 1/Hevin in pancreatic cancer. Neoplasia 9: 8–17. 17325739 10.1593/neo.06646 PMC1803032

[61] Vafadar-Isfahani B, Ball G, Coveney C, Lemetre C, Boocock D, et al (2012) Identification of SPARC-like 1 protein as part of a biomarker panel for Alzheimer's disease in cerebrospinal fluid. J Alzheimers Dis 28: 625–636. 10.3233/JAD-2011-111505 22045497

[62] Turtoi A, Musmeci D, Naccarato AG, Scatena C, Ortenzi V, et al (2012) Sparc-like protein 1 is a new marker of human glioma progression. J Proteome Res 11: 5011–5021. 10.1021/pr3005698 22909274

[63] Yamanaka M, Kanda K, Li NC, Fukumori T, Oka N, et al (2001) Analysis of the gene expression of SPARC and its prognostic value for bladder cancer. J Urol 166: 2495–2499. 11696817

[64] Yin F, Liu X, Li D, Wang Q, Zhang W, et al (2013) Bioinformatic analysis of chemokine (C-C motif) ligand 21 and SPARC-like protein 1 revealing their associations with drug resistance in ovarian cancer. Int J Oncol 42: 1305–1316. 10.3892/ijo.2013.1819 23404140

[65] He P, He HZ, Dai J, Wang Y, Sheng QH, et al (2005) The human plasma proteome: analysis of Chinese serum using shotgun strategy. Proteomics 5: 3442–3453. 16047309 10.1002/pmic.200401301

[66] Myklebust MP, Fluge O, Immervoll H, Skarstein A, Balteskard L, et al (2012) Expression of DSG1 and DSC1 are prognostic markers in anal carcinoma patients. Br J Cancer 106: 756–762. 10.1038/bjc.2011.548 22333708 PMC3322941

[67] Caruso JA, Stemmer PM (2011) Proteomic profiling of lipid rafts in a human breast cancer model of tumorigenic progression. Clin Exp Metastasis 28: 529–540. 10.1007/s10585-011-9389-5 21533873 PMC3827680

[68] Black MH, Diamandis EP (2000) The diagnostic and prognostic utility of prostate-specific antigen for diseases of the breast. Breast Cancer Res Treat 59: 1–14. 10752675 10.1023/a:1006380306781

[69] Rittenhouse HG, Finlay JA, Mikolajczyk SD, Partin AW (1998) Human Kallikrein 2 (hK2) and prostate-specific antigen (PSA): two closely related, but distinct, kallikreins in the prostate. Crit Rev Clin Lab Sci 35: 275–368. 9759557 10.1080/10408369891234219

[70] Sloan KE, Eustace BK, Stewart JK, Zehetmeier C, Torella C, et al (2004) CD155/PVR plays a key role in cell motility during tumor cell invasion and migration. BMC Cancer 4: 73 15471548 10.1186/1471-2407-4-73 PMC524493

[71] Pavlova NN, Pallasch C, Elia AE, Braun CJ, Westbrook TF, et al (2013) A role for PVRL4-driven cell-cell interactions in tumorigenesis. Elife 2: e00358 10.7554/eLife.00358 23682311 PMC3641523

[72] Descamps S, Lebourhis X, Delehedde M, Boilly B, Hondermarck H (1998) Nerve growth factor is mitogenic for cancerous but not normal human breast epithelial cells. J Biol Chem 273: 16659–16662. 9642218 10.1074/jbc.273.27.16659

[73] Dolle L, Adriaenssens E, El Yazidi-Belkoura I, Le Bourhis X, Nurcombe V, et al (2004) Nerve growth factor receptors and signaling in breast cancer. Curr Cancer Drug Targets 4: 463–470. 15379632 10.2174/1568009043332853

[74] Nutter F, Holen I, Brown H, Cross S, Evans A, et al (2014) Different molecular profiles are associated with breast cancer bone homing compared to colonisation. Endocr Relat Cancer. 10.1530/ERC-13-0158 24413608

[75] Lee KM, Nam K, Oh S, Lim J, Kim YP, et al (2014) Extracellular matrix protein 1 regulates cell proliferation and trastuzumab resistance through activation of epidermal growth factor signaling. Breast Cancer Res 16: 479 10.1186/s13058-014-0479-6 25499743 PMC4308848

[76] Han Z, Ni J, Smits P, Underhill CB, Xie B, et al (2001) Extracellular matrix protein 1 (ECM1) has angiogenic properties and is expressed by breast tumor cells. Faseb j 15: 988–994. 11292659 10.1096/fj.99-0934com

[77] Wang L, Yu J, Ni J, Xu XM, Wang J, et al (2003) Extracellular matrix protein 1 (ECM1) is over-expressed in malignant epithelial tumors. Cancer Lett 200: 57–67. 14550953 10.1016/s0304-3835(03)00350-1

[78] Lal G, Hashimi S, Smith BJ, Lynch CF, Zhang L, et al (2009) Extracellular matrix 1 (ECM1) expression is a novel prognostic marker for poor long-term survival in breast cancer: a Hospital-based Cohort Study in Iowa. Ann Surg Oncol 16: 2280–2287. 10.1245/s10434-009-0533-2 19521735

[79] Wu QW, She HQ, Liang J, Huang YF, Yang QM, et al (2012) Expression and clinical significance of extracellular matrix protein 1 and vascular endothelial growth factor-C in lymphatic metastasis of human breast cancer. BMC Cancer 12: 47 10.1186/1471-2407-12-47 22284579 PMC3292501

[80] Zhu DJ, Chen XW, Wang JZ, Ju YL, Ou Yang MZ, et al (2013) Proteomic analysis identifies proteins associated with curcumin-enhancing efficacy of irinotecan-induced apoptosis of colorectal cancer LOVO cell. Int J Clin Exp Pathol 7: 1–15. 24427321 PMC3885455

[81] Antalis CJ, Uchida A, Buhman KK, Siddiqui RA (2011) Migration of MDA-MB-231 breast cancer cells depends on the availability of exogenous lipids and cholesterol esterification. Clin Exp Metastasis 28: 733–741. 10.1007/s10585-011-9405-9 21744083

[82] dos Santos CR, Domingues G, Matias I, Matos J, Fonseca I, et al (2014) LDL-cholesterol signaling induces breast cancer proliferation and invasion. Lipids Health Dis 13: 16 10.1186/1476-511X-13-16 24428917 PMC3896822

[83] Coffelt SB, de Visser KE (2015) Immune-mediated mechanisms influencing the efficacy of anticancer therapies. Trends Immunol 36: 198–216. 10.1016/j.it.2015.02.006 25857662

[84] Coffelt SB, Kersten K, Doornebal CW, Weiden J, Vrijland K, et al (2015) IL-17-producing gammadelta T cells and neutrophils conspire to promote breast cancer metastasis. Nature. 10.1038/nature14282 PMC4475637 25822788

[85] Gyorffy B, Lanczky A, Eklund AC, Denkert C, Budczies J, et al (2010) An online survival analysis tool to rapidly assess the effect of 22,277 genes on breast cancer prognosis using microarray data of 1,809 patients. Breast Cancer Res Treat 123: 725–731. 10.1007/s10549-009-0674-9 20020197

