# Supplementary figures and images for: Proteomic Analysis of Urine to Identify Breast Cancer Biomarker Candidates Using a Label-Free LC-MS/MS Approach
Source: PLoS One. 2015 Nov 6;10(11):e0141876. doi: 10.1371/journal.pone.0141876 (PMC4636393; doi:10.1371/journal.pone.0141876)

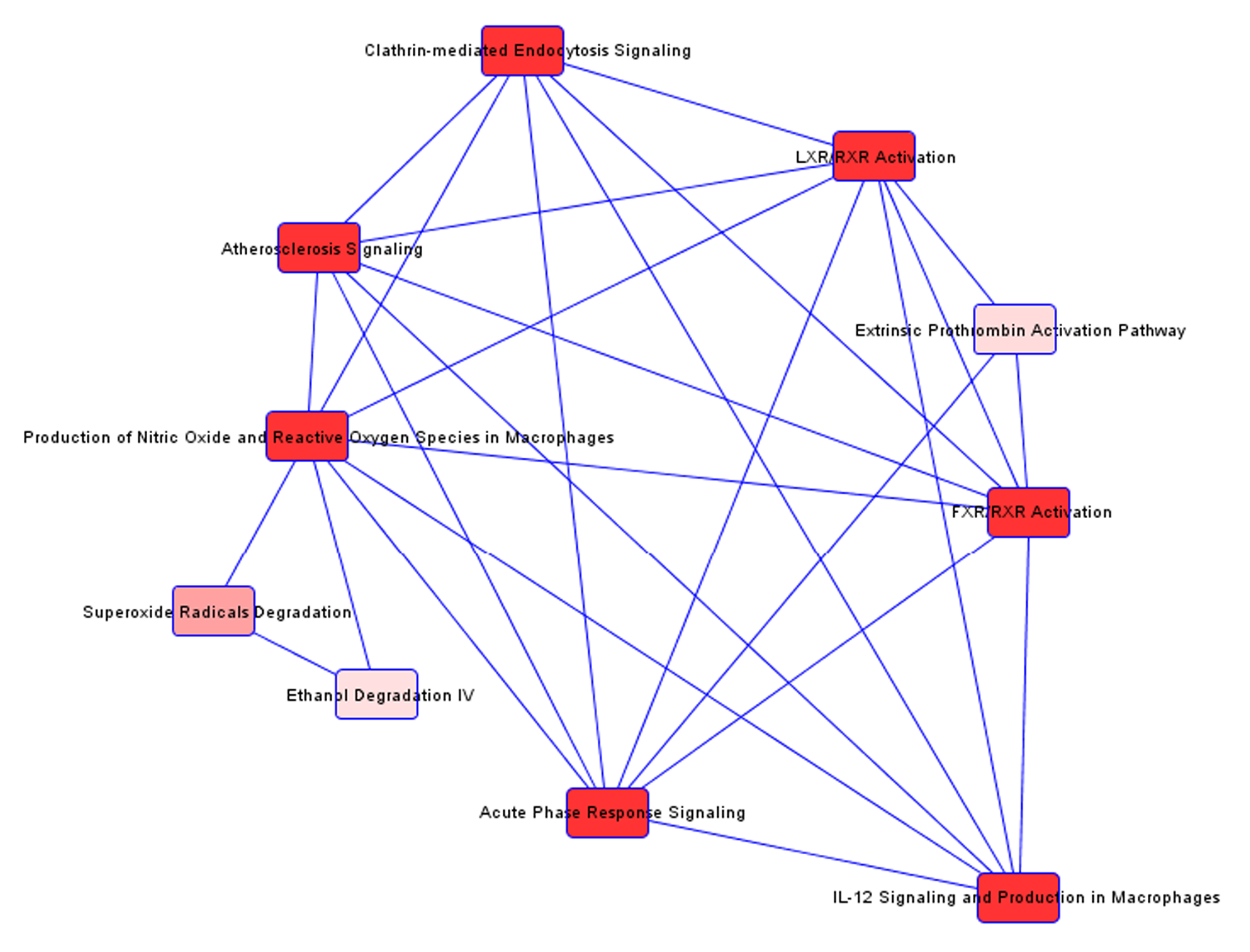

Supplement: S1 Fig — (TIFF) [file pone.0141876.s001.tiff]
