# Supplementary material for: Proteomic Analysis of Urine to Identify Breast Cancer Biomarker Candidates Using a Label-Free LC-MS/MS Approach
Source: PLoS One. 2015 Nov 6;10(11):e0141876. doi: 10.1371/journal.pone.0141876 (PMC4636393; doi:10.1371/journal.pone.0141876)
Supplement: S1 Table — (DOCX) [file pone.0141876.s002.docx]

| Human Access.  ID | Uni-Prot  ID | Protein Description | Peptides  ID | Score | Fold | SL | BC Ref. | Non BC Ref. |
| --- | --- | --- | --- | --- | --- | --- | --- | --- |
|  |  | ***DCIS*** |  |  |  |  |  |  |
| *ENOA* | **P06733** | Alpha-enolase ^Ř^ | 3 | 85 | 7.8 | C | [[1-3](#_ENREF_1)] | [[4](#_ENREF_4)] |
| *KNG1* | **P01042** | Kininogen-1 ^Ř^ | 9 | 469 | 3.6 | S | [[5](#_ENREF_5)] | [[6](#_ENREF_6), [7](#_ENREF_7)] |
| *SCG1* | **P05060** | Secretogranin-1 ^Đ^ | 4 | 178 | 8.2 | S |  | [[8](#_ENREF_8)] |
|  |  |  |  |  |  |  |  |  |
|  |  | ***IBC*** |  |  |  |  |  |  |
| *AMPN* | **P15144** | Aminopeptidase N ^Ř^ | 5 | 228 | 3.1 | C | [[9](#_ENREF_9), [10](#_ENREF_10)] | [[11-13](#_ENREF_11)] |
| *DCD* | **P81605** | Dermcidin^Ř^ | 2 | 117 | 4.9 | S | [[14](#_ENREF_14)] | [[15](#_ENREF_15)] |
| *DSG1* | **Q02413** | Desmoglein-1 ^Đ^ | 2 | 55 | 3.0 | S |  | [[16](#_ENREF_16)] |
| *KLK1* | **P06870** | Kallikrein-1 ^Đ^ | 1 | 71 | 3.2 | S | HKK2-3 [[17](#_ENREF_17), [18](#_ENREF_18)] KLK5 [[19](#_ENREF_19)] KLK12-13 [[20](#_ENREF_20), [21](#_ENREF_21)] |  |
| *K22E* | **P35908** | Keratin, type II cytoskeletal 2 epidermal | 27 | 1619 | 5.0 | C |  |  |
| *PVR* | **P15151** | Poliovirus receptor ^Đ^ | 2 | 86 | 3.3 | CM & S | PVRL4 [[22](#_ENREF_22)], [[23](#_ENREF_23)] | [[24](#_ENREF_24)] |
|  |  |  |  |  |  |  |  |  |
|  |  | ***MBC*** |  |  |  |  |  |  |
| *TRFE (+1)* | **P02787** | Serotransferrin^Ř^ | 12 | 511 | 3.8 | S | [[25](#_ENREF_25), [26](#_ENREF_26)] |  |
| *VASN* | **Q6EMK4** | Vasorin^Đ^ | 2 | 80 | 5.5 | CM |  | [[27](#_ENREF_27)] |
| *VMO1* | **Q7Z5L0** | Vitelline membrane outer layer protein 1 homolog | 3 | 110 | 5.4 | S |  |  |

*Notes:* **Accession ID**, Human accession identification; **Uni-Prot ID**, Protein identification based on the Protein knowledge base UniProtKB/Swiss-Prot ID (<http://www.uniprot.org>); **Peptides ID**, Assigned peptides identified; **Score**, Mascot score; **SL**, Sub-cellular location as annotated in UniProtKB. **Fold**: Fold change for BC samples against control. The proteins of interest showing biological significance are underlined. For ease of navigation, all proteins reported in the literature in association with BC were marked ^Ŕ^ or with other disease marked ^Đ^. Plasma Proteins detected in Normal Urine*[[28](#_ENREF_28)]. *SL Abbreviations:* C, Cytoplasm; CM, Cell membrane; S, Secreted.

**REFERENCES for S1 Table.**

1. Tu SH, Chang CC, Chen CS, Tam KW, Wang YJ, Lee CH, et al. Increased expression of enolase alpha in human breast cancer confers tamoxifen resistance in human breast cancer cells. Breast cancer research and treatment. 2010;121(3):539-53. Epub 2009/08/06. doi: 10.1007/s10549-009-0492-0. PubMed PMID: 19655245.

2. Zamani-Ahmadmahmudi M, Nassiri SM, Rahbarghazi R. Serological proteome analysis of dogs with breast cancer unveils common serum biomarkers with human counterparts. Electrophoresis. 2013. Epub 2013/12/18. doi: 10.1002/elps.201300461. PubMed PMID: 24338489.

3. Shih NY, Lai HL, Chang GC, Lin HC, Wu YC, Liu JM, et al. Anti-alpha-enolase autoantibodies are down-regulated in advanced cancer patients. Japanese journal of clinical oncology. 2010;40(7):663-9. Epub 2010/04/17. doi: 10.1093/jjco/hyq028. PubMed PMID: 20395242.

4. Liu Z, Chen C, Yang H, Zhang Y, Long J, Long X, et al. Proteomic features of potential tumor suppressor NESG1 in nasopharyngeal carcinoma. Proteomics. 2012;12(22):3416-25. Epub 2012/09/22. doi: 10.1002/pmic.201200146. PubMed PMID: 22997098.

5. Kim BK, Lee JW, Park PJ, Shin YS, Lee WY, Lee KA, et al. The multiplex bead array approach to identifying serum biomarkers associated with breast cancer. Breast Cancer Res. 2009;11(2):R22. Epub 2009/04/30. doi: 10.1186/bcr2247. PubMed PMID: 19400944; PubMed Central PMCID: PMCPmc2688951.

6. Wang J, Wang X, Lin S, Chen C, Wang C, Ma Q, et al. Identification of kininogen-1 as a serum biomarker for the early detection of advanced colorectal adenoma and colorectal cancer. PLoS One. 2013;8(7):e70519. Epub 2013/07/31. doi: 10.1371/journal.pone.0070519. PubMed PMID: 23894665; PubMed Central PMCID: PMCPmc3720899.

7. Liu W, Liu B, Cai Q, Li J, Chen X, Zhu Z. Proteomic identification of serum biomarkers for gastric cancer using multi-dimensional liquid chromatography and 2D differential gel electrophoresis. Clinica chimica acta; international journal of clinical chemistry. 2012;413(13-14):1098-106. Epub 2012/03/27. doi: 10.1016/j.cca.2012.03.003. PubMed PMID: 22446497.

8. Yang MS, Wang HS, Wang BS, Li WH, Pang ZF, Zou BK, et al. A comparative proteomic study identified calreticulin and prohibitin up-regulated in adrenocortical carcinomas. Diagnostic pathology. 2013;8:58. Epub 2013/04/17. doi: 10.1186/1746-1596-8-58. PubMed PMID: 23587357; PubMed Central PMCID: PMCPMC3640901.

9. Liang X, Zhao J, Hajivandi M, Wu R, Tao J, Amshey JW, et al. Quantification of membrane and membrane-bound proteins in normal and malignant breast cancer cells isolated from the same patient with primary breast carcinoma. J Proteome Res. 2006;5(10):2632-41. Epub 2006/10/07. doi: 10.1021/pr060125o. PubMed PMID: 17022634.

10. Ranogajec I, Jakic-Razumovic J, Puzovic V, Gabrilovac J. Prognostic value of matrix metalloproteinase-2 (MMP-2), matrix metalloproteinase-9 (MMP-9) and aminopeptidase N/CD13 in breast cancer patients. Medical oncology (Northwood, London, England). 2012;29(2):561-9. Epub 2011/05/26. doi: 10.1007/s12032-011-9984-y. PubMed PMID: 21611838.

11. Hashida H, Takabayashi A, Kanai M, Adachi M, Kondo K, Kohno N, et al. Aminopeptidase N is involved in cell motility and angiogenesis: its clinical significance in human colon cancer. Gastroenterology. 2002;122(2):376-86. Epub 2002/02/08. PubMed PMID: 11832452.

12. Zoidakis J, Makridakis M, Zerefos PG, Bitsika V, Esteban S, Frantzi M, et al. Profilin 1 is a potential biomarker for bladder cancer aggressiveness. Mol Cell Proteomics. 2012;11(4):M111 009449. Epub 2011/12/14. doi: 10.1074/mcp.M111.009449. PubMed PMID: 22159600; PubMed Central PMCID: PMCPMC3322560.

13. Surowiak P, Drag M, Materna V, Suchocki S, Grzywa R, Spaczynski M, et al. Expression of aminopeptidase N/CD13 in human ovarian cancers. International journal of gynecological cancer : official journal of the International Gynecological Cancer Society. 2006;16(5):1783-8. Epub 2006/10/03. doi: 10.1111/j.1525-1438.2006.00657.x. PubMed PMID: 17009972.

14. Porter D, Weremowicz S, Chin K, Seth P, Keshaviah A, Lahti-Domenici J, et al. A neural survival factor is a candidate oncogene in breast cancer. Proceedings of the National Academy of Sciences of the United States of America. 2003;100(19):10931-6. Epub 2003/09/04. doi: 10.1073/pnas.1932980100. PubMed PMID: 12953101; PubMed Central PMCID: PMCPmc196905.

15. Stewart GD, Skipworth RJ, Pennington CJ, Lowrie AG, Deans DA, Edwards DR, et al. Variation in dermcidin expression in a range of primary human tumours and in hypoxic/oxidatively stressed human cell lines. Br J Cancer. 2008;99(1):126-32. Epub 2008/07/03. doi: 10.1038/sj.bjc.6604458. PubMed PMID: 18594538; PubMed Central PMCID: PMCPmc2453008.

16. Myklebust MP, Fluge O, Immervoll H, Skarstein A, Balteskard L, Bruland O, et al. Expression of DSG1 and DSC1 are prognostic markers in anal carcinoma patients. Br J Cancer. 2012;106(4):756-62. Epub 2012/02/16. doi: 10.1038/bjc.2011.548. PubMed PMID: 22333708; PubMed Central PMCID: PMCPmc3322941.

17. Black MH, Diamandis EP. The diagnostic and prognostic utility of prostate-specific antigen for diseases of the breast. Breast cancer research and treatment. 2000;59(1):1-14. Epub 2000/04/07. PubMed PMID: 10752675.

18. Rittenhouse HG, Finlay JA, Mikolajczyk SD, Partin AW. Human Kallikrein 2 (hK2) and prostate-specific antigen (PSA): two closely related, but distinct, kallikreins in the prostate. Critical reviews in clinical laboratory sciences. 1998;35(4):275-368. Epub 1998/10/06. doi: 10.1080/10408369891234219. PubMed PMID: 9759557.

19. Avgeris M, Papachristopoulou G, Polychronis A, Scorilas A. Down-regulation of kallikrein-related peptidase 5 (KLK5) expression in breast cancer patients: a biomarker for the differential diagnosis of breast lesions. Clinical proteomics. 2011;8(1):5. Epub 2011/09/13. doi: 10.1186/1559-0275-8-5. PubMed PMID: 21906360; PubMed Central PMCID: PMCPmc3167201.

20. Yousef GM, Magklara A, Diamandis EP. KLK12 is a novel serine protease and a new member of the human kallikrein gene family-differential expression in breast cancer. Genomics. 2000;69(3):331-41. Epub 2000/11/01. doi: 10.1006/geno.2000.6346. PubMed PMID: 11056051.

21. Yousef GM, Chang A, Diamandis EP. Identification and characterization of KLK-L4, a new kallikrein-like gene that appears to be down-regulated in breast cancer tissues. J Biol Chem. 2000;275(16):11891-8. Epub 2000/04/15. PubMed PMID: 10766816.

22. Pavlova NN, Pallasch C, Elia AE, Braun CJ, Westbrook TF, Hemann M, et al. A role for PVRL4-driven cell-cell interactions in tumorigenesis. eLife. 2013;2:e00358. Epub 2013/05/18. doi: 10.7554/eLife.00358. PubMed PMID: 23682311; PubMed Central PMCID: PMCPMC3641523.

23. Fabre-Lafay S, Monville F, Garrido-Urbani S, Berruyer-Pouyet C, Ginestier C, Reymond N, et al. Nectin-4 is a new histological and serological tumor associated marker for breast cancer. BMC Cancer. 2007;7:73. Epub 2007/05/04. doi: 10.1186/1471-2407-7-73. PubMed PMID: 17474988; PubMed Central PMCID: PMCPMC1868744.

24. Sloan KE, Eustace BK, Stewart JK, Zehetmeier C, Torella C, Simeone M, et al. CD155/PVR plays a key role in cell motility during tumor cell invasion and migration. BMC Cancer. 2004;4:73. Epub 2004/10/09. doi: 10.1186/1471-2407-4-73. PubMed PMID: 15471548; PubMed Central PMCID: PMCPMC524493.

25. Somiari RI, Sullivan A, Russell S, Somiari S, Hu H, Jordan R, et al. High-throughput proteomic analysis of human infiltrating ductal carcinoma of the breast. Proteomics. 2003;3(10):1863-73. Epub 2003/11/20. doi: 10.1002/pmic.200300560. PubMed PMID: 14625848.

26. Zeng Z, Hincapie M, Haab BB, Hanash S, Pitteri SJ, Kluck S, et al. The development of an integrated platform to identify breast cancer glycoproteome changes in human serum. J Chromatogr A. 2010;1217(19):3307-15. Epub 2009/09/29. doi: 10.1016/j.chroma.2009.09.029. PubMed PMID: 19782370.

27. Loftheim H, Midtvedt K, Hartmann A, Reisaeter AV, Falck P, Holdaas H, et al. Urinary proteomic shotgun approach for identification of potential acute rejection biomarkers in renal transplant recipients. Transplantation research. 2012;1(1):9. Epub 2013/02/02. doi: 10.1186/2047-1440-1-9. PubMed PMID: 23369437; PubMed Central PMCID: PMCPmc3561036.

28. Candiano G, Santucci L, Petretto A, Bruschi M, Dimuccio V, Urbani A, et al. 2D-electrophoresis and the urine proteome map: where do we stand? Journal of Proteomics. 2010;73(5):829-44. PubMed PMID: 20004755.
