# Supplementary material for: Proteomic Analysis of Urine to Identify Breast Cancer Biomarker Candidates Using a Label-Free LC-MS/MS Approach
Source: PLoS One. 2015 Nov 6;10(11):e0141876. doi: 10.1371/journal.pone.0141876 (PMC4636393; doi:10.1371/journal.pone.0141876)
Supplement: S2 Table — (DOCX) [file pone.0141876.s003.docx]

| Human  Access.ID | Uni-Prot  ID | Protein Description | SL | Peptides  ID | Score | DCIS  FC | IBC  FC | MBC  FC | BBD  FC | BC Ref. | Non BC  Ref. |
| --- | --- | --- | --- | --- | --- | --- | --- | --- | --- | --- | --- |
|  |  | ***Up-regulated in DCIS*** |  |  |  |  |  |  |  |  |  |
| *APOA1* | **P02647** | Apolipoprotein A-I^Ř^ | S | 2 | 103 | ↑5.5 | ↓4 |  |  | [[1-5](#_ENREF_1)] | [[6-10](#_ENREF_6)]. |
| *ECM1* | **Q16610** | Extracellular matrix protein 1 ^Ř^ | S | 3 | 69 | ↑13 | ↑30 |  |  | [[11](#_ENREF_11), [12](#_ENREF_12)] |  |
| *KV118* | **P01610** | Ig kappa chain V-I region WEA | MF | 5 | 392 | ↑8 | ↑10 |  |  |  |  |
| *LAC2* | **P0CG05** | Ig lambda-2 chain C regions* | MF | 5 | 246 | ↑5 | ↑4 |  | ↑11 |  |  |
| *TTHY* | **P02766** | Transthyretin*^Ř^ | S, C | 3 | 142 | ↑97 |  |  | ↓38 | [[4](#_ENREF_4), [13](#_ENREF_13), [14](#_ENREF_14)] | [[10](#_ENREF_10), [15-17](#_ENREF_15)] |
|  |  | ***Up-regulated in IBC*** |  |  |  |  |  |  |  |  |  |
| *A1BG* | **P04217** | Alpha-1B-glycoprotein* ^Ř^ | S | 2 | 104 | ↓8 | ↑14 | ↑10 |  | [[18](#_ENREF_18)] | [[19](#_ENREF_19), [20](#_ENREF_20)] |
| *CATA* | **P04040** | Catalase ^Ř^ | CO | 1 | 57 |  | ↑4 |  | ↑5 | [[21-23](#_ENREF_21)] |  |
| *CO6A1* | **P12109** | Collagen alpha-1(VI) chain*^Ř^ | S | 2 | 114 |  | ↑4.5 | ↑7 |  | [[24](#_ENREF_24)] CO6A2 [[25](#_ENREF_25)] | [[26](#_ENREF_26), [27](#_ENREF_27)] |
| *FILA* | **P20930** | Filaggrin ^Đ^ | N | 1 | 60 |  | ↑32 |  | ↓11 |  | [[28](#_ENREF_28), [29](#_ENREF_29)] |
| *HV303* | **P01764** | Ig heavy chain V-III region VH26 | MF | 2 | 80 |  | ↑41 |  | ↑6 |  |  |
| *MMRN2* | **Q9H8L6** | Multimerin-2 ^Đ^ | S | 1 | 34 | ↓43 | ↑6 |  |  |  | [[30](#_ENREF_30), [31](#_ENREF_31)] |
| *PI16* | **Q6UXB8** | Peptidase inhibitor 16 ^Đ^ | M | 2 | 92 | ↓4 | ↑9.5 |  | ↑15 |  | [[32](#_ENREF_32), [33](#_ENREF_33)] |
| *AMBP* | **P02760** | Protein AMBP ^Ř^ | S | 11 | 664 |  | ↑11 |  | ↑4 | [[3](#_ENREF_3), [34](#_ENREF_34)] | [[35](#_ENREF_35)] |
| *CD014* | **Q8NC60** | Uncharacterized protein C4orf14 | M | 3 | 78 |  | ↑228 |  | ↓12 |  |  |
|  |  | ***Up-regulated in MBC*** |  |  |  |  |  |  |  |  |  |
| *FIBA* | **P02671** | Fibrinogen alpha chain* ^Đ^ | S | 3 | 248 | ↓4 | ↓5 | ↑3 | ND | FIBG [[36](#_ENREF_36)] |  |
| *K1C10* | **P13645** | Keratin, type I cytoskeletal 10 ^Đ^ | C | 25 | 1475 | ND | ↓5 | ↑4 | ↓3 | K1C 16,18- 19 [[25](#_ENREF_25), [37-39](#_ENREF_37)] | [[40](#_ENREF_40)] |
|  |  | ***Up-regulated in BBD*** |  |  |  |  |  |  |  |  |  |
| *CADH1* | **P12830** | Cadherin-1 ^Ř^ | CM | 2 | 83 | ↓5 |  |  | ↑4 | [[41](#_ENREF_41)]  E-cadherin [[42](#_ENREF_42)] |  |
| *KV122* | **P04430** | Ig kappa chain V-I region BAN | MF | 1 | 64 | ↓21 |  |  | ↑3 |  |  |
|  |  | ***Down-regulated in DCIS*** |  |  |  |  |  |  |  |  |  |
| *FILA2* | **Q5D862** | Filaggrin-2 ^Đ^ | U | 2 | 88 | ↓3 | ↓5 |  |  |  | [[28](#_ENREF_28)] |
| *HORN* | **Q86YZ3** | Hornerin ^Đ^ | C | 1 | 51 | ↓8 | ↓15 |  |  |  | [[29](#_ENREF_29), [43](#_ENREF_43)] |
| *IGHG4* | **P01861** | Ig gamma-4 chain C region | S | 4 | 142 | ↓12 |  |  | ↓12 |  |  |
| *NUCB1* | **Q02818** | Nucleobindin-1 ^Đ^ | C, M | 1 | 32 | ↓11 |  |  | ↓200 |  | [[44](#_ENREF_44)] |
| *TLR4* | **O00206** | Toll-like receptor 4 ^Ř^ | M | 3 | 83 | ↓43 | ↓7 |  | ↓4 | [[45-48](#_ENREF_45)] |  |
|  |  | ***Down-regulated IBC & BBD*** |  |  |  |  |  |  |  |  |  |
| *PLAK* | **P14923** | Junction plakoglobin ^Ř^ | C, M | 2 | 77 |  | ↓7 |  | ↓5 | [[49](#_ENREF_49), [50](#_ENREF_50)] |  |
| *MASP2* | **O00187** | Mannan-binding lectin serine protease 2^Đ^ | S | 2 | 86 |  | ↓10 |  | ↓32 |  | [[51-53](#_ENREF_51)] |
|  |  |  |  |  |  |  |  |  |  |  |  |

*Notes:* The expression patterns of the various proteins either up-regulated (↑) or down regulated (↓), demonstrate an important relationship between the different stages of BC. **Accession ID**, Human accession identification; **Uni-Prot ID**, Protein identification based on the Protein knowledge base UniProtKB/Swiss-Prot ID (<http://www.uniprot.org>); **Peptides ID**, Assigned Peptides Identified; **Score**, Mascot score; **SL**, Sub-cellular location as annotated in UniProtKB. **FC**: Fold change for BC samples against control. The proteins of interest showing biological significance are underlined. All proteins reported in the literature ^Ř^ indicate that these proteins have been reported to be associated with BC or associated with other cancers ^Đ^. Plasma Proteins detected in Normal Urine*[[54](#_ENREF_54)]. *SL Abbreviations:* C, Cytoplasm; CM, Cell membrane; CO, Cell organelle; M, Membrane; MF, Membrane fraction; N, Nucleus; S, Secreted.

**REFERENCES for S2 Table.**

1. Hamrita B, Ben Nasr H, Gabbouj S, Bouaouina N, Chouchane L, Chahed K. Apolipoprotein A1 -75 G/A and +83 C/T polymorphisms: susceptibility and prognostic implications in breast cancer. Molecular biology reports. 2011;38(3):1637-43. Epub 2010/09/16. doi: 10.1007/s11033-010-0274-0. PubMed PMID: 20842444.

2. Meng R, Gormley M, Bhat VB, Rosenberg A, Quong AA. Low abundance protein enrichment for discovery of candidate plasma protein biomarkers for early detection of breast cancer. J Proteomics. 2011;75(2):366-74. Epub 2011/08/20. doi: 10.1016/j.jprot.2011.07.030. PubMed PMID: 21851866.

3. Cho W, Jung K, Regnier FE. Sialylated Lewis x antigen bearing glycoproteins in human plasma. J Proteome Res. 2010;9(11):5960-8. Epub 2010/09/23. doi: 10.1021/pr100747p. PubMed PMID: 20858014; PubMed Central PMCID: PMCPMC2976037.

4. Goncalves A, Esterni B, Bertucci F, Sauvan R, Chabannon C, Cubizolles M, et al. Postoperative serum proteomic profiles may predict metastatic relapse in high-risk primary breast cancer patients receiving adjuvant chemotherapy. Oncogene. 2006;25(7):981-9. Epub 2005/09/28. doi: 10.1038/sj.onc.1209131. PubMed PMID: 16186794.

5. Kim BK, Lee JW, Park PJ, Shin YS, Lee WY, Lee KA, et al. The multiplex bead array approach to identifying serum biomarkers associated with breast cancer. Breast Cancer Res. 2009;11(2):R22. Epub 2009/04/30. doi: 10.1186/bcr2247. PubMed PMID: 19400944; PubMed Central PMCID: PMCPmc2688951.

6. Loftheim H, Midtvedt K, Hartmann A, Reisaeter AV, Falck P, Holdaas H, et al. Urinary proteomic shotgun approach for identification of potential acute rejection biomarkers in renal transplant recipients. Transplantation research. 2012;1(1):9. Epub 2013/02/02. doi: 10.1186/2047-1440-1-9. PubMed PMID: 23369437; PubMed Central PMCID: PMCPmc3561036.

7. Lei T, Zhao X, Jin S, Meng Q, Zhou H, Zhang M. Discovery of potential bladder cancer biomarkers by comparative urine proteomics and analysis. Clin Genitourin Cancer. 2013;11(1):56-62. Epub 2012/09/18. doi: 10.1016/j.clgc.2012.06.003. PubMed PMID: 22982111.

8. Chen CL, Lin TS, Tsai CH, Wu CC, Chung T, Chien KY, et al. Identification of potential bladder cancer markers in urine by abundant-protein depletion coupled with quantitative proteomics. J Proteomics. 2013;85:28-43. Epub 2013/05/02. doi: 10.1016/j.jprot.2013.04.024. PubMed PMID: 23631828.

9. Chen Y-T, Chen C-L, Chen H-W, Chung T, Wu C-C, Chen C-D, et al. Discovery of novel bladder cancer biomarkers by comparative urine proteomics using iTRAQ technology. Journal of Proteome Research. 2010;9(11):5803-15. PubMed PMID: 20806971.

10. Zhang Z, Bast RC, Jr., Yu Y, Li J, Sokoll LJ, Rai AJ, et al. Three biomarkers identified from serum proteomic analysis for the detection of early stage ovarian cancer. Cancer Res. 2004;64(16):5882-90. Epub 2004/08/18. doi: 10.1158/0008-5472.can-04-0746. PubMed PMID: 15313933.

11. Xiong GP, Zhang JX, Gu SP, Wu YB, Liu JF. Overexpression of ECM1 contributes to migration and invasion in cholangiocarcinoma cell. Neoplasma. 2012;59(4):409-15. Epub 2012/04/12. doi: 10.4149/neo_2012_053. PubMed PMID: 22489696.

12. Nutter F, Holen I, Brown H, Cross S, Evans A, Walker M, et al. Different molecular profiles are associated with breast cancer bone homing compared to colonisation. Endocrine-related cancer. 2014. Epub 2014/01/15. doi: 10.1530/erc-13-0158. PubMed PMID: 24413608.

13. Nasim FU, Ejaz S, Ashraf M, Asif AR, Oellerich M, Ahmad G, et al. Potential biomarkers in the sera of breast cancer patients from bahawalpur, pakistan. Biomarkers in cancer. 2012;4:19-34. Epub 2012/01/01. doi: 10.4137/bic.s10502. PubMed PMID: 24179392; PubMed Central PMCID: PMCPMC3791917.

14. Majidzadeh AK, Gharechahi J. Plasma proteomics analysis of tamoxifen resistance in breast cancer. Medical oncology (Northwood, London, England). 2013;30(4):753. Epub 2013/10/26. doi: 10.1007/s12032-013-0753-y. PubMed PMID: 24158757.

15. Sigdel TK, Lau K, Schilling J, Sarwal M. Optimizing protein recovery for urinary proteomics, a tool to monitor renal transplantation. Clinical Transplantation. 2008;22(5):617-23. PubMed PMID: 18459997.

16. Dekker LJ, Boogerd W, Stockhammer G, Dalebout JC, Siccama I, Zheng P, et al. MALDI-TOF mass spectrometry analysis of cerebrospinal fluid tryptic peptide profiles to diagnose leptomeningeal metastases in patients with breast cancer. Mol Cell Proteomics. 2005;4(9):1341-9. Epub 2005/06/23. doi: 10.1074/mcp.M500081-MCP200. PubMed PMID: 15970584.

17. Rompp A, Dekker L, Taban I, Jenster G, Boogerd W, Bonfrer H, et al. Identification of leptomeningeal metastasis-related proteins in cerebrospinal fluid of patients with breast cancer by a combination of MALDI-TOF, MALDI-FTICR and nanoLC-FTICR MS. Proteomics. 2007;7(3):474-81. Epub 2007/02/03. doi: 10.1002/pmic.200600719. PubMed PMID: 17274072.

18. Zeng Z, Hincapie M, Haab BB, Hanash S, Pitteri SJ, Kluck S, et al. The development of an integrated platform to identify breast cancer glycoproteome changes in human serum. J Chromatogr A. 2010;1217(19):3307-15. Epub 2009/09/29. doi: 10.1016/j.chroma.2009.09.029. PubMed PMID: 19782370.

19. Soggiu A, Piras C, Bonizzi L, Hussein HA, Pisanu S, Roncada P. A discovery-phase urine proteomics investigation in type 1 diabetes. Acta diabetologica. 2012;49(6):453-64. Epub 2012/06/09. doi: 10.1007/s00592-012-0407-0. PubMed PMID: 22678621.

20. Kreunin P, Zhao J, Rosser C, Urquidi V, Lubman DM, Goodison S. Bladder cancer associated glycoprotein signatures revealed by urinary proteomic profiling. J Proteome Res. 2007;6(7):2631-9. Epub 2007/05/24. doi: 10.1021/pr0700807. PubMed PMID: 17518487; PubMed Central PMCID: PMCPmc2668245.

21. Panis C, Victorino VJ, Herrera AC, Freitas LF, De Rossi T, Campos FC, et al. Differential oxidative status and immune characterization of the early and advanced stages of human breast cancer. Breast cancer research and treatment. 2012;133(3):881-8. Epub 2011/11/04. doi: 10.1007/s10549-011-1851-1. PubMed PMID: 22048816.

22. Yeghiazaryan K, Mamlouk S, Trog D, Moenkemann H, Braun M, Kuhn W, et al. Irradiated breast cancer patients demonstrate subgroup-specific regularities in protein expression patterns of circulating leukocytes. Cancer genomics & proteomics. 2007;4(6):411-8. Epub 2008/01/22. PubMed PMID: 18204204.

23. Glorieux C, Dejeans N, Sid B, Beck R, Calderon PB, Verrax J. Catalase overexpression in mammary cancer cells leads to a less aggressive phenotype and an altered response to chemotherapy. Biochemical pharmacology. 2011;82(10):1384-90. Epub 2011/06/22. doi: 10.1016/j.bcp.2011.06.007. PubMed PMID: 21689642.

24. Abba MC, Drake JA, Hawkins KA, Hu Y, Sun H, Notcovich C, et al. Transcriptomic changes in human breast cancer progression as determined by serial analysis of gene expression. Breast Cancer Res. 2004;6(5):R499-513. Epub 2004/08/21. doi: 10.1186/bcr899. PubMed PMID: 15318932; PubMed Central PMCID: PMCPmc549167.

25. Yi W, Peng J, Zhang Y, Fu F, Zou Q, Tang Y. [Differential protein expressions in breast cancer between drug sensitive tissues and drug resistant tissues]. Zhong nan da xue xue bao Yi xue ban = Journal of Central South University Medical sciences. 2013;38(2):148-54. Epub 2013/03/05. doi: 10.3969/j.issn.1672-7347.2013.02.007. PubMed PMID: 23456065.

26. Fan NJ, Gao CF, Wang CS, Zhao G, Lv JJ, Wang XL, et al. Identification of the up-regulation of TP-alpha, collagen alpha-1(VI) chain, and S100A9 in esophageal squamous cell carcinoma by a proteomic method. J Proteomics. 2012;75(13):3977-86. Epub 2012/05/16. doi: 10.1016/j.jprot.2012.05.008. PubMed PMID: 22583932.

27. Chaudhary N, Bhatnagar S, Malik S, Katare DP, Jain SK. Proteomic analysis of differentially expressed proteins in lung cancer in Wistar rats using NNK as an inducer. Chemico-biological interactions. 2013;204(2):125-34. Epub 2013/05/23. doi: 10.1016/j.cbi.2013.05.004. PubMed PMID: 23692979.

28. Scharenberg C, Eckardt A, Tiede C, Kreipe H, Hussein K. Expression of caspase 14 and filaggrin in oral squamous carcinoma. Head and neck pathology. 2013;7(4):327-33. Epub 2013/05/07. doi: 10.1007/s12105-013-0445-0. PubMed PMID: 23645350; PubMed Central PMCID: PMCPmc3824792.

29. Pellerin L, Henry J, Hsu CY, Balica S, Jean-Decoster C, Mechin MC, et al. Defects of filaggrin-like proteins in both lesional and nonlesional atopic skin. The Journal of allergy and clinical immunology. 2013;131(4):1094-102. Epub 2013/02/14. doi: 10.1016/j.jaci.2012.12.1566. PubMed PMID: 23403047.

30. Soltermann A, Ossola R, Kilgus-Hawelski S, von Eckardstein A, Suter T, Aebersold R, et al. N-glycoprotein profiling of lung adenocarcinoma pleural effusions by shotgun proteomics. Cancer. 2008;114(2):124-33. Epub 2008/03/11. doi: 10.1002/cncr.23349. PubMed PMID: 18327805.

31. Shield-Artin KL, Bailey MJ, Oliva K, Liovic AK, Barker G, Dellios NL, et al. Identification of ovarian cancer-associated proteins in symptomatic women: A novel method for semi-quantitative plasma proteomics. Proteomics Clinical applications. 2012;6(3-4):170-81. Epub 2012/04/26. doi: 10.1002/prca.201100008. PubMed PMID: 22532453.

32. Freue GV, Sasaki M, Meredith A, Gunther OP, Bergman A, Takhar M, et al. Proteomic signatures in plasma during early acute renal allograft rejection. Mol Cell Proteomics. 2010;9(9):1954-67. Epub 2010/05/27. doi: 10.1074/mcp.M110.000554. PubMed PMID: 20501940; PubMed Central PMCID: PMCPMC2938106.

33. Reeves JR, Dulude H, Panchal C, Daigneault L, Ramnani DM. Prognostic value of prostate secretory protein of 94 amino acids and its binding protein after radical prostatectomy. Clinical cancer research : an official journal of the American Association for Cancer Research. 2006;12(20 Pt 1):6018-22. Epub 2006/10/26. doi: 10.1158/1078-0432.ccr-06-0625. PubMed PMID: 17062675.

34. Cohen A, Wang E, Chisholm KA, Kostyleva R, O'Connor-McCourt M, Pinto DM. A mass spectrometry-based plasma protein panel targeting the tumor microenvironment in patients with breast cancer. J Proteomics. 2013;81:135-47. Epub 2012/11/24. doi: 10.1016/j.jprot.2012.11.004. PubMed PMID: 23174118.

35. Braoudaki M, Lambrou GI, Vougas K, Karamolegou K, Tsangaris GT, Tzortzatou-Stathopoulou F. Protein biomarkers distinguish between high- and low-risk pediatric acute lymphoblastic leukemia in a tissue specific manner. Journal of hematology & oncology. 2013;6:52. Epub 2013/07/16. doi: 10.1186/1756-8722-6-52. PubMed PMID: 23849470; PubMed Central PMCID: PMCPMC3717072.

36. Dirix LY, Salgado R, Weytjens R, Colpaert C, Benoy I, Huget P, et al. Plasma fibrin D-dimer levels correlate with tumour volume, progression rate and survival in patients with metastatic breast cancer. Br J Cancer. 2002;86(3):389-95. Epub 2002/03/05. doi: 10.1038/sj.bjc.6600069. PubMed PMID: 11875705; PubMed Central PMCID: PMCPmc2375200.

37. Rower C, Koy C, Hecker M, Reimer T, Gerber B, Thiesen HJ, et al. Mass spectrometric characterization of protein structure details refines the proteome signature for invasive ductal breast carcinoma. Journal of the American Society for Mass Spectrometry. 2011;22(3):440-56. PubMed PMID: 21472563.

38. Somiari RI, Sullivan A, Russell S, Somiari S, Hu H, Jordan R, et al. High-throughput proteomic analysis of human infiltrating ductal carcinoma of the breast. Proteomics. 2003;3(10):1863-73. Epub 2003/11/20. doi: 10.1002/pmic.200300560. PubMed PMID: 14625848.

39. Rezaul K, Thumar JK, Lundgren DH, Eng JK, Claffey KP, Wilson L, et al. Differential protein expression profiles in estrogen receptor-positive and -negative breast cancer tissues using label-free quantitative proteomics. Genes & cancer. 2010;1(3):251-71. Epub 2010/03/01. doi: 10.1177/1947601910365896. PubMed PMID: 21779449; PubMed Central PMCID: PMCPmc3092194.

40. Chen J, Cheng X, Merched-Sauvage M, Caulin C, Roop DR, Koch PJ. An unexpected role for keratin 10 end domains in susceptibility to skin cancer. Journal of cell science. 2006;119(Pt 24):5067-76. Epub 2006/11/23. doi: 10.1242/jcs.03298. PubMed PMID: 17118961.

41. Wendt MK, Taylor MA, Schiemann BJ, Schiemann WP. Down-regulation of epithelial cadherin is required to initiate metastatic outgrowth of breast cancer. Molecular biology of the cell. 2011;22(14):2423-35. Epub 2011/05/27. doi: 10.1091/mbc.E11-04-0306. PubMed PMID: 21613543; PubMed Central PMCID: PMCPmc3135469.

42. Qureshi HS, Linden MD, Divine G, Raju UB. E-cadherin status in breast cancer correlates with histologic type but does not correlate with established prognostic parameters. Am J Clin Pathol. 2006;125(3):377-85. Epub 2006/04/15. PubMed PMID: 16613340.

43. Fleming JM, Ginsburg E, Oliver SD, Goldsmith P, Vonderhaar BK. Hornerin, an S100 family protein, is functional in breast cells and aberrantly expressed in breast cancer. BMC Cancer. 2012;12:266. Epub 2012/06/26. doi: 10.1186/1471-2407-12-266. PubMed PMID: 22727333; PubMed Central PMCID: PMCPmc3464886.

44. Zhu DJ, Chen XW, Wang JZ, Ju YL, Ou Yang MZ, Zhang WJ. Proteomic analysis identifies proteins associated with curcumin-enhancing efficacy of irinotecan-induced apoptosis of colorectal cancer LOVO cell. International journal of clinical and experimental pathology. 2013;7(1):1-15. Epub 2014/01/16. PubMed PMID: 24427321; PubMed Central PMCID: PMCPmc3885455.

45. Basith S, Manavalan B, Yoo TH, Kim SG, Choi S. Roles of toll-like receptors in cancer: a double-edged sword for defense and offense. Archives of pharmacal research. 2012;35(8):1297-316. Epub 2012/09/04. doi: 10.1007/s12272-012-0802-7. PubMed PMID: 22941474.

46. Gonzalez-Reyes S, Marin L, Gonzalez L, Gonzalez LO, del Casar JM, Lamelas ML, et al. Study of TLR3, TLR4 and TLR9 in breast carcinomas and their association with metastasis. BMC Cancer. 2010;10:665. Epub 2010/12/07. doi: 10.1186/1471-2407-10-665. PubMed PMID: 21129170; PubMed Central PMCID: PMCPMC3009680.

47. Yang H, Zhou H, Feng P, Zhou X, Wen H, Xie X, et al. Reduced expression of Toll-like receptor 4 inhibits human breast cancer cells proliferation and inflammatory cytokines secretion. Journal of experimental & clinical cancer research : CR. 2010;29:92. Epub 2010/07/14. doi: 10.1186/1756-9966-29-92. PubMed PMID: 20618976; PubMed Central PMCID: PMCPMC2913950.

48. Theodoropoulos GE, Saridakis V, Karantanos T, Michalopoulos NV, Zagouri F, Kontogianni P, et al. Toll-like receptors gene polymorphisms may confer increased susceptibility to breast cancer development. Breast (Edinburgh, Scotland). 2012;21(4):534-8. Epub 2012/05/09. doi: 10.1016/j.breast.2012.04.001. PubMed PMID: 22560646.

49. Mukhina S, Mertani HC, Guo K, Lee KO, Gluckman PD, Lobie PE. Phenotypic conversion of human mammary carcinoma cells by autocrine human growth hormone. Proceedings of the National Academy of Sciences of the United States of America. 2004;101(42):15166-71. Epub 2004/09/09. doi: 10.1073/pnas.0405881101. PubMed PMID: 15353581; PubMed Central PMCID: PMCPmc524067.

50. Holen I, Whitworth J, Nutter F, Evans A, Brown HK, Lefley DV, et al. Loss of plakoglobin promotes decreased cell-cell contact, increased invasion, and breast cancer cell dissemination in vivo. Breast Cancer Res. 2012;14(3):R86. Epub 2012/05/29. doi: 10.1186/bcr3201. PubMed PMID: 22632416; PubMed Central PMCID: PMCPmc3446349.

51. Alves G, Pereira DA, Sandim V, Ornellas AA, Escher N, Melle C, et al. Urine screening by Seldi-Tof, followed by biomarker identification, in a Brazilian cohort of patients with renal cell carcinoma (RCC). International braz j urol : official journal of the Brazilian Society of Urology. 2013;39(2):228-39. Epub 2013/05/21. doi: 10.1590/s1677-5538.ibju.2013.02.12. PubMed PMID: 23683669.

52. Fisch U, Zehnder A, Hirt A, Niggli F, Simon A, Ozsahin H, et al. Mannan-binding lectin (MBL) and MBL-associated serine protease-2 in children with cancer. Swiss medical weekly. 2011;141:w13191. Epub 2011/04/30. doi: 10.4414/smw.2011.13191. PubMed PMID: 21528466.

53. Ytting H, Christensen IJ, Thiel S, Jensenius JC, Nielsen HJ. Serum mannan-binding lectin-associated serine protease 2 levels in colorectal cancer: relation to recurrence and mortality. Clinical cancer research : an official journal of the American Association for Cancer Research. 2005;11(4):1441-6. Epub 2005/03/05. doi: 10.1158/1078-0432.ccr-04-1272. PubMed PMID: 15746044.

54. Candiano G, Santucci L, Petretto A, Bruschi M, Dimuccio V, Urbani A, et al. 2D-electrophoresis and the urine proteome map: where do we stand? Journal of Proteomics. 2010;73(5):829-44. PubMed PMID: 20004755.
