# Supplementary material for: Proteomic Analysis of Urine to Identify Breast Cancer Biomarker Candidates Using a Label-Free LC-MS/MS Approach
Source: PLoS One. 2015 Nov 6;10(11):e0141876. doi: 10.1371/journal.pone.0141876 (PMC4636393; doi:10.1371/journal.pone.0141876)
Supplement: S3 Table — (DOCX) [file pone.0141876.s004.docx]

| HumanAccess.ID | Uni-Prot  ID | Protein Description | Peptides  ID | Score | Fold | SL | BC Ref. | Non BC Ref. |
| --- | --- | --- | --- | --- | --- | --- | --- | --- |
|  |  | ***BBD up-regulated*** |  |  |  |  |  |  |
| *KV309* | **P04433** | Ig kappa chain V-III region VG (Fragment) | 1 | 35 | 1070 | MF |  |  |
| *IGLL5* | **B9A064** | Immunoglobulin lambda-like polypeptide 5 | 4 | 157 | 3.6 | S |  |  |
| *F184A* | **Q8NB25** | Protein FAM184A ^Đ^ | 3 | 75 | 3.6 | C |  |  |
| *E2F8* | **A0AVK6** | Transcription factor E2F8 ^Đ^ | 3 | 102 | 3.9 | N | E2F-1 [[1](#_ENREF_1)], E2F-4 [[2](#_ENREF_2)] | [[3](#_ENREF_3), [4](#_ENREF_4)] |
|  |  | ***BBD down regulated*** |  |  |  |  |  |  |
| *ADML* | **P35318** | ADM (Adrenomedullin) ^Ř^ | 1 | 46 | 10. 6 | S | [[5](#_ENREF_5), [6](#_ENREF_6)] | [[7](#_ENREF_7)] |
| *A2ML1* | **A8K2U0** | Alpha-2-macroglobulin-like protein 1^Ř^ | 2 | 84 | 16.0 | S | [[8](#_ENREF_8), [9](#_ENREF_9)] |  |
| *CERU* | **P00450** | Ceruloplasmin* ^Đ^ | 9 | 382 | 4.8 | S |  | [[10](#_ENREF_10)] |
| *DMKN* | **Q6E0U4** | Dermokine^Đ^ | 1 | 56 | 4.1 | S |  | [[11-13](#_ENREF_11)] |
| *HPT* | **P00738** | Haptoglobin*^Đ^ | 1 | 65 | 3.4 | S |  | [[14](#_ENREF_14), [15](#_ENREF_15)] |
| *KV203* | **P01616** | Ig kappa chain V-II region MIL | 1 | 75 | 37.9 | MF |  |  |
| *K1C9* | **P35527** | Keratin, type I cytoskeletal 9 | 27 | 1660 | 3.2 | C | K1C9 [[16](#_ENREF_16)] K1C18-19[[17-19](#_ENREF_17)] |  |
| *SPRR3* | **Q9UBC9** | Small proline-rich protein 3 ^Ř^ | 13 | 450 | 4.0 | C | [[20](#_ENREF_20)] | [[21](#_ENREF_21)] |
| *THBG* | **P05543** | Thyroxine-binding globulin | 3 | 154 | 5.6 | S |  |  |
|  |  |  |  |  |  |  |  |  |

*Notes:* **Accession ID**, Human Accession identification; **Uni-Prot ID**, Protein identification based on the Protein knowledge base UniProtKB/Swiss-Prot ID (<http://www.uniprot.org>); **Pep ID**, Assigned Peptides Identified; **Score**, Mascot score; **SL**, Sub-cellular location as annotated in UniProtKB. **Fold**: Fold change for BC samples against control. The proteins of interest showing biological significance are underlined. For ease of navigation, all proteins reported in the literature in association with BC are marked ^Ŕ^ or with other disease marked ^Đ^. Plasma Proteins detected in Normal Urine*[[22](#_ENREF_22)].

*SL Abbreviations:* C, Cytoplasm; MF, Membrane fraction; N, Nucleus.

**REFERENCES for S3 Table.**

1. Zacharatos P, Kotsinas A, Evangelou K, Karakaidos P, Vassiliou LV, Rezaei N, et al. Distinct expression patterns of the transcription factor E2F-1 in relation to tumour growth parameters in common human carcinomas. The Journal of pathology. 2004;203(3):744-53. Epub 2004/06/29. doi: 10.1002/path.1582. PubMed PMID: 15221933.

2. Rakha EA, Pinder SE, Paish EC, Robertson JF, Ellis IO. Expression of E2F-4 in invasive breast carcinomas is associated with poor prognosis. The Journal of pathology. 2004;203(3):754-61. Epub 2004/06/29. doi: 10.1002/path.1573. PubMed PMID: 15221934.

3. Deng Q, Wang Q, Zong WY, Zheng DL, Wen YX, Wang KS, et al. E2F8 contributes to human hepatocellular carcinoma via regulating cell proliferation. Cancer Res. 2010;70(2):782-91. Epub 2010/01/14. doi: 10.1158/0008-5472.can-09-3082. PubMed PMID: 20068156.

4. Xanthoulis A, Tiniakos DG. E2F transcription factors and digestive system malignancies: how much do we know? World journal of gastroenterology : WJG. 2013;19(21):3189-98. Epub 2013/06/08. doi: 10.3748/wjg.v19.i21.3189. PubMed PMID: 23745020; PubMed Central PMCID: PMCPmc3671070.

5. Oehler MK, Fischer DC, Orlowska-Volk M, Herrle F, Kieback DG, Rees MC, et al. Tissue and plasma expression of the angiogenic peptide adrenomedullin in breast cancer. Br J Cancer. 2003;89(10):1927-33. Epub 2003/11/13. doi: 10.1038/sj.bjc.6601397. PubMed PMID: 14612905; PubMed Central PMCID: PMCPmc2394432.

6. Martinez A, Vos M, Guedez L, Kaur G, Chen Z, Garayoa M, et al. The effects of adrenomedullin overexpression in breast tumor cells. Journal of the National Cancer Institute. 2002;94(16):1226-37. Epub 2002/08/22. PubMed PMID: 12189226.

7. Nikitenko LL, Leek R, Henderson S, Pillay N, Turley H, Generali D, et al. The G-protein-coupled receptor CLR is upregulated in an autocrine loop with adrenomedullin in clear cell renal cell carcinoma and associated with poor prognosis. Clinical cancer research : an official journal of the American Association for Cancer Research. 2013;19(20):5740-8. Epub 2013/08/24. doi: 10.1158/1078-0432.ccr-13-1712. PubMed PMID: 23969937; PubMed Central PMCID: PMCPmc3836221.

8. Opstal-van Winden AW, Krop EJ, Karedal MH, Gast MC, Lindh CH, Jeppsson MC, et al. Searching for early breast cancer biomarkers by serum protein profiling of pre-diagnostic serum; a nested case-control study. BMC Cancer. 2011;11:381. Epub 2011/08/30. doi: 1471-2407-11-381 [pii]

10.1186/1471-2407-11-381 [doi]. PubMed PMID: 21871081.

9. Kadowaki M, Sangai T, Nagashima T, Sakakibara M, Yoshitomi H, Takano S, et al. Identification of vitronectin as a novel serum marker for early breast cancer detection using a new proteomic approach. Journal of cancer research and clinical oncology. 2011;137(7):1105-15. Epub 2011/01/22. doi: 10.1007/s00432-010-0974-9. PubMed PMID: 21253761.

10. Peng L, Liu J, Li YM, Huang ZL, Wang PP, Gu YR, et al. Serum proteomics analysis and comparisons using iTRAQ in the progression of hepatitis B. Experimental and therapeutic medicine. 2013;6(5):1169-76. Epub 2013/11/14. doi: 10.3892/etm.2013.1310. PubMed PMID: 24223640; PubMed Central PMCID: PMCPmc3820766.

11. Watanabe K, Oochiai T, Kikuchi S, Kumano T, Matsui T, Morimoto K, et al. Dermokine expression in intraductal papillary-mucinous neoplasm and invasive pancreatic carcinoma. Anticancer Res. 2012;32(10):4405-12. Epub 2012/10/13. PubMed PMID: 23060565.

12. Tagi T, Matsui T, Kikuchi S, Hoshi S, Ochiai T, Kokuba Y, et al. Dermokine as a novel biomarker for early-stage colorectal cancer. Journal of gastroenterology. 2010;45(12):1201-11. Epub 2010/07/24. doi: 10.1007/s00535-010-0279-4. PubMed PMID: 20652332.

13. Szabo GT, Tihanyi R, Csulak F, Jambor E, Bona A, Szabo G, et al. Comparative salivary proteomics of cleft palate patients. The Cleft palate-craniofacial journal : official publication of the American Cleft Palate-Craniofacial Association. 2012;49(5):519-23. Epub 2011/04/21. doi: 10.1597/10-135. PubMed PMID: 21504360.

14. Bharti A, Ma PC, Maulik G, Singh R, Khan E, Skarin AT, et al. Haptoglobin alpha-subunit and hepatocyte growth factor can potentially serve as serum tumor biomarkers in small cell lung cancer. Anticancer Res. 2004;24(2c):1031-8. Epub 2004/05/25. PubMed PMID: 15154618.

15. Kwak JY, Ma TZ, Yoo MJ, Choi BH, Kim HG, Kim SR, et al. The comparative analysis of serum proteomes for the discovery of biomarkers for acute myeloid leukemia. Experimental hematology. 2004;32(9):836-42. Epub 2004/09/04. doi: 10.1016/j.exphem.2004.06.006. PubMed PMID: 15345285.

16. Yi W, Peng J, Zhang Y, Fu F, Zou Q, Tang Y. [Differential protein expressions in breast cancer between drug sensitive tissues and drug resistant tissues]. Zhong nan da xue xue bao Yi xue ban = Journal of Central South University Medical sciences. 2013;38(2):148-54. Epub 2013/03/05. doi: 10.3969/j.issn.1672-7347.2013.02.007. PubMed PMID: 23456065.

17. Rezaul K, Thumar JK, Lundgren DH, Eng JK, Claffey KP, Wilson L, et al. Differential protein expression profiles in estrogen receptor-positive and -negative breast cancer tissues using label-free quantitative proteomics. Genes & cancer. 2010;1(3):251-71. Epub 2010/03/01. doi: 10.1177/1947601910365896. PubMed PMID: 21779449; PubMed Central PMCID: PMCPmc3092194.

18. Rower C, Koy C, Hecker M, Reimer T, Gerber B, Thiesen HJ, et al. Mass spectrometric characterization of protein structure details refines the proteome signature for invasive ductal breast carcinoma. Journal of the American Society for Mass Spectrometry. 2011;22(3):440-56. PubMed PMID: 21472563.

19. Somiari RI, Sullivan A, Russell S, Somiari S, Hu H, Jordan R, et al. High-throughput proteomic analysis of human infiltrating ductal carcinoma of the breast. Proteomics. 2003;3(10):1863-73. Epub 2003/11/20. doi: 10.1002/pmic.200300560. PubMed PMID: 14625848.

20. Kim JC, Yu JH, Cho YK, Jung CS, Ahn SH, Gong G, et al. Expression of SPRR3 is associated with tumor cell proliferation in less advanced stages of breast cancer. Breast cancer research and treatment. 2012;133(3):909-16. Epub 2011/11/15. doi: 10.1007/s10549-011-1868-5. PubMed PMID: 22076481.

21. Cho DH, Jo YK, Roh SA, Na YS, Kim TW, Jang SJ, et al. Upregulation of SPRR3 promotes colorectal tumorigenesis. Molecular medicine (Cambridge, Mass). 2010;16(7-8):271-7. Epub 2010/04/10. doi: 10.2119/molmed.2009.00187. PubMed PMID: 20379613; PubMed Central PMCID: PMCPmc2896463.

22. Candiano G, Santucci L, Petretto A, Bruschi M, Dimuccio V, Urbani A, et al. 2D-electrophoresis and the urine proteome map: where do we stand? Journal of Proteomics. 2010;73(5):829-44. PubMed PMID: 20004755.
